# Regulatory role of lncH19 in RAC1 alternative splicing: implication for RAC1B expression in colorectal cancer

**DOI:** 10.1186/s13046-024-03139-z

**Published:** 2024-08-05

**Authors:** Aurora Cordaro, Maria Magdalena Barreca, Chiara Zichittella, Marco Loria, Denise Anello, Goffredo Arena, Nicolina Sciaraffa, Claudia Coronnello, Giuseppe Pizzolanti, Riccardo Alessandro, Alice Conigliaro

**Affiliations:** 1 Department of Biomedicine Neuroscience and Advanced Diagnostic, University of Palermo, Palermo, Italy; 2 McGill University Health Centre, Montréal, Canada; 3 Fondazione Istituto G. Giglio di Cefalù, Cefalù, Italy; 4 Advanced Data Analysis Group, Ri.MED Foundation, Palermo, Italy; 5 Dipartimento di Promozione della Salute, Materno-Infantile, di Medicina Interna e Specialistica di Eccellenza “G. D’Alessandro”, PROMISE, University of Palermo, Palermo, 90127 Italy; 6 AteN Center-Advanced Technologies Network Center, University of Palermo, Palermo, 90128 Italy; 7 Institute for Biomedical Research and Innovation (IRIB), National Research Council (CNR), Palermo, Italy

**Keywords:** lncH19, Colorectal cancer, Alternative splicing, RNA-binding proteins, RBFOX2, RAC1

## Abstract

Aberrant alternative splicing events play a critical role in cancer biology, contributing to tumor invasion, metastasis, epithelial-mesenchymal transition, and drug resistance. Recent studies have shown that alternative splicing is a key feature for transcriptomic variations in colorectal cancer, which ranks third among malignant tumors worldwide in both incidence and mortality. Long non-coding RNAs can modulate this process by acting as trans-regulatory agents, recruiting splicing factors, or driving them to specific targeted genes. LncH19 is a lncRNA dis-regulated in several tumor types and, in colorectal cancer, it plays a critical role in tumor onset, progression, and metastasis. In this paper, we found, that in colorectal cancer cells, the long non-coding RNA H19 can bind immature RNAs and splicing factors as hnRNPM and RBFOX2. Through bioinformatic analysis, we identified 57 transcripts associated with lncH19 and containing binding sites for both splicing factors, hnRNPM, and RBFOX2. Among these transcripts, we identified the mRNA of the GTPase-RAC1, whose alternatively spliced isoform, RAC1B, has been ascribed several roles in the malignant transformation. We confirmed, in vitro, the binding of the splicing factors to both the transcripts RAC1 and lncH19. Loss and gain of expression experiments in two colorectal cancer cell lines (SW620 and HCT116) demonstrated that lncH19 is required for RAC1B expression and, through RAC1B, it induces c-Myc and Cyclin-D increase. In vivo, investigation from biopsies of colorectal cancer patients showed higher levels of all the explored genes (lncH19, RAC1B, c-Myc and Cyclin-D) concerning the healthy counterpart, thus supporting our in vitro model. In addition, we identified a positive correlation between lncH19 and RAC1B in colorectal cancer patients. Finally, we demonstrated that lncH19, as a shuttle, drives the splicing factors RBFOX2 and hnRNPM to RAC1 allowing exon retention and RAC1B expression. The data shown in this paper represent the first evidence of a new mechanism of action by which lncH19 carries out its functions as an oncogene by prompting colorectal cancer through the modulation of alternative splicing.

**Supplementary Information:**

The online version contains supplementary material available at 10.1186/s13046-024-03139-z.

## Introduction

According to the World Health Organization, in 2020, there were over 1.9 million new cases of colorectal cancer (CRC) worldwide, leading to more than 930,000 deaths. Despite advancements in surgical strategies, chemotherapy, and early diagnosis, CRC is still the third most common cancer and the second leading cause of cancer-related deaths globally. More efforts should be made to understand tumor behaviour and the molecular mechanisms involved in cancer progression.

Basic research highlighted the pivotal role of many non-coding RNAs in carcinogenesis so that some of them are counted among oncogenes and their expression is considered a biomarker for bad prognosis [1]. H19 is the first lncRNA found to be overexpressed in gastric cancers and hepatocellular carcinoma, subsequently identified as deregulated also in different types of cancer i.e. breast cancer, osteosarcoma, pancreatic cancers, esophageal squamous cell carcinoma, and colorectal cancer (see [2] and included references). LncH19 expression is associated with cancer cell proliferation, Epithelial to Mesenchymal Transition (EMT), and drug resistance [3]. Largely investigated from its discovery to date, the data in the literature indicated that lncH19 can affect the development of cancer by working as a sponge for miRNAs, miRNAs precursor, and epigenetic modulator through the interaction with EZH2 and Polycomb complex [2, 4]. No data have been yet collected about a possible role of lncH19 in Alternative splicing (AS).

The AS is an important post-transcriptional regulatory mechanism, over 95% of human genes undergo alternative splicing and emerging data demonstrated that aberrant AS events were closely associated with cancer progression, metastasis, therapeutic resistance, and other oncogenic processes [5]. In CRC, AS is a key feature for transcriptomic variations, likely to be an important determinant of both prognosis and biological regulation of cancer [6, 7]. The inclusion or exclusion of an exon in the mature transcript depends on both cis-regulatory sequences, present in the pre-mRNA, and trans-regulatory agents. These cis-regulatory sequences fall into four categories, i.e., Intronic Splicing Enhancers (ISEs), Intronic Splicing Silencers (ISSs), Exonic Splicing Enhancers (ESEs), and Exonic Splicing Silencers (ESSs). To control the splicing process, these sequences bind to various regulatory proteins, including heterogeneous nuclear ribonucleoproteins (hnRNPs), SR (Serine-Arginine rich) proteins, RBFOX1 and RBFOX2. These RNA binding proteins (RBPs) regulate mRNA splicing and are called splicing factors (SFs) [8].

RBFOX2 is the master regulator of tissue-specific alternative splicing, implicated in the development of ovarian and breast cancer as well as in EMT [9–11]. RBFOX2 recognizes different sets of alternatively spliced RNAs, sometimes also without a specific binding motif thanks to its interaction with multiple proteins [12, 13]. More recently it has been demonstrated its implication in the microexon splicing, associated with CRC metastasis [14]. Overall, the data in the literature attributed to RBFOX2 a role as an inductor of oncogenic splice-switching that drives an invasive phenotype in cancer.

The body of experimental evidence supporting the connection between long non-coding RNAs (lncRNAs) and alternative splicing (AS) in cancers is growing. Research has shown that the deregulation of lncRNAs can be both the result and the cause of AS. In the last case, the non-coding RNAs work together with splicing factors to act as a trans-regulatory agent and play a role in pro-tumorigenic AS processes [15].

It has been found that deregulation of certain lncRNA is associated with AS in colorectal cancer (CRC) [16]. However, as far as we know, there is no available data on whether lncH19 plays a role in AS in general or in CRC specifically.

The data here described demonstrated that lncH19 interacts with the splicing factors RBFOX2 and brings this to specific splicing sites. In particular, we demonstrated that lncH19 is required for the AS of RAC1 and the expression of RAC1B, a constitutively activated GTPase [17] whose expression in human colorectal cancer is associated with aggressive disease and poor prognosis [18].

## Materials and methods

### Cell culture

Human SW620 and HCT116 colorectal cancer cells (ATCC–LGC Standards S.r.L., Italy) were cultured respectively in RPMI and McCoy’s 5 A medium (Euroclone, United Kingdom) supplemented with 10% fetal bovine serum (FBS, Euroclone, United Kingdom), 1% penicillin/streptomycin (10.000 U/mL penicillin and 10 mg/mL streptomycin), and 200 mM L-glutamine (all from Euroclone). Cells were maintained in a humidified 5% CO2 atmosphere at 37 °C and used at early passages for all the experiments. The culture medium was changed every 2–3 days, and cells were split at 70–80% of confluence. Depending on the experimental setup, treatment with the RAC1 inhibitor EHT1864 (cat n° SC-361175, Santa Cruz Biotechnology), was done as follows: 24 h after seeding in 12wells multiwell plates, cells were treated with 50mM of EHT1864 for 18 h; then the cells were processed for the following experiments.

### Patient’s tissue samples

Twenty paired tissues (CRC and adjacent non-tumor) were collected from patients who were diagnosed with CRC at Giglio Hospital (Cefalù, Palermo, Italy). All patients underwent no preoperative therapy before surgical resection and have signed informed consent. The use of the collected samples was approved by the Institutional Ethics Committee of Palermo 1/Fondazione Giglio Cefalù prot 246/2020. The specimens collected from the patients were preserved in RNAlaterTM (cat. n°. AM7021 Thermo Fisher^®^ Scientific, United States) for subsequent RNA extraction and gene expression analysis.

### Cell transfection to overexpress lncH19

SW620 and HCT116 cells were seeded respectively at 3 × 10^4^ or 2.5 × 10^4^ per cm^2^. After 24 h, cells were transfected with 1.2 µg/ml of H19-pFLAG-CMV-2 expression vector (cat. n° E7033, Sigma-Aldrich, USA), or empty pFLAG-CMV-2 as Negative Control (Sigma-Aldrich). For cell transfection, HiPerFect Transfection Reagent (cat. n° 301704, Qiagen, Germany) was used following the manufacturer’s standard instructions. Eighteen hours after transfection, the cells were processed for the following experiments.

### Infection with lentiviral vectors to stably silence lncH19

SW620 and HCT116 cells were stably silenced for lncH19 by lentiviral infection with H19 human shRNA lentiviral particles (cat. n° TL318197V, OriGene Technologies, Inc., United States), relative control cells were infected with control shRNA lentiviral particles (cat. n° TR30021V, OriGene Technologies, Inc., United States). The infected cells were selected by cell sorting (BD FACSAria™ III Sorter, ATeN Center, Italy) and maintained under selective pressure with 1 mg/mL of puromycin (Gibco™ puromycin dihydrochloride, cat. n°A1113802, Thermo Fisher^®^ Scientific). QRT-PCR and fluorescence microscopy regularly tested H19 silencing efficiency.

### Cell transfection to silence RBFOX2

SW620 and HCT116 cells were seeded respectively at 3 × 10^4^ or 2.5 × 10^4^ per cm^2^. After 24 h, cells were transfected with two different silencer^®^ Pre-designed siRNA for RBFOX2 (siRNA ID respectively 136602 and 136602 form Life Technologies), or siRNA scramble (scr) as Negative Control. For cell transfection, HiPerFect Transfection Reagent (cat. n° 301704, Qiagen, Germany) was used following the manufacturer’s standard instructions. Eighteen hours after transfection, the cells were processed for the following experiments.

### RNA extraction and real-time PCR (qRT-PCR)

Total RNA was isolated from the cells by using a commercially available miRNA purification Kit (NucleoSpin™ miRNA kit, cat. n° 740971.250, Macherey–Nagel, Germany), according to the manufacturer’s instructions. The total RNA concentration was detected with the Nanodrop spectrophotometer (Thermo Fisher^®^) and reverse-transcribed to cDNA using the High-Capacity cDNA Reverse Transcription kit (cat. n° 4368814, Applied Biosystem™, United States).

Quantitative real-time polymerase chain reactions (qRT-PCR) were performed by using the SYBR™ Green PCR Master Mix (cat. n° 4309155, Applied Biosystems™), following the manufacturer’s instructions in a Step One™ Real-time PCR System Thermal Cycling Block (Applied Biosystems™). The primers’ sequences used for gene expression analysis are reported in Table 1. The relative expression of mRNAs was analyzed using the 2^−ΔΔCt^ method with β-actin or 28 S serving as internal reference genes.

**Table 1:** The sequence of the primers used for gene expression analysis

Gene	Primer Forward (5’-3’)	Primer Reverse (5’-3’)
H19	TCG​TGC​AGA​CAG​GGC​GAC​ATC	CCA​GCT​GCC​ACG​TCC​TGT​AAC​C
β-ACTIN	CAAGAGATGGCCACGGCTGCT	TCCTTCTGCATCCTGTCGGCA
28 S	CCGTGCCTTGGAAAGCGTCGC	CAGAGGCTGTTCACCTTGGAGA
RAC1	AAACCGGTGAATCTGGGCTT	AAGAACACATCTGTTTGCGGA
RAC1B	AAACCGGTGAATCTGGGCTT	ATCGGCAATCGGCTTGTCTT
CYCLIN D1	AAAGAATTTGCACCCCGCTG	GACAGACAAAGCGTCCCTCA
cMYC	TACAACACCCGAGCAAGGAC	CTAACGTTGAGGGGCATCGT
rbFOX2	CCAGCTTTCAAGCAGATGTGTCC	CAAATGGGCTCCTCTGAAAGCG

### RNA antisense precipitation (RAP) to investigate lncH19-protein interaction

SW620 or HCT116 cells were harvested and lysed in a specific polysome lysis buffer (10 mM Tris-HCl, 20 mM KCl, 1.5 mM MgCl_2_, 0.5% Nonidet P-40, 5 mM DTT, 5% Glycerol, 40 U/ml RNase inhibitor) on ice for 1.30 h. Cell debris was removed by centrifugation at 15.000 x *g* for 10 min at 4 °C and the supernatant, containing the protein lysate, was quantified through the Bradford microassay method (cat. n° 1856210 ThermoFisher) using bovine serum albumin (BSA, Sigma-Aldrich) as a standard. For each assay, a total of 500 µg of proteins, were incubated with 100pmol of biotin-labeled H19 probes or control probes (designed from Sigma-Aldrich) to pull down lncH19-protein complexes, for 30 min at room temperature. Streptavidin-labeled magnetic beads were then added to the samples and incubated for 30 min at room temperature to precipitate lncH19-proteins-probe-coated beads. After washing the beads with TENT Buffer (20 mM Tris-HCl pH8.0, 2 mM EDTA pH 8.0, 500 mM NaCl, 1% Triton X-100) the lncH19-protein complex was eluted by incubating the beads with elution buffer (0.5 M NH_4_OH, 0.5 mM EDTA) 30 min at 4 °C. The protein complexes bound to the beads were denatured by heating at 70 °C for 10 min and analyzed by Immunoblotting (See Table 2).

**Table 2:** The sequence of the biotin-labeled H19 probes used for RNA pull-down assays

Oligo name	Sequences (5’-3’)
comH19 var 1–1	GTGCAGCATATTCATTTCCA
comH19 var 1–2	GTTTTGTGTCCGGATTCAAAGG
comH19 var 1–3	TGCCCCTGTGCCTGCTACTAAAT
comH19 var 1–4	TCCTGCCAGACTCCAGATGT
comH19 var 1–5	CTTCCCCAGTTTCCCCCGTTACC
comH19 var 1-scr1	GAAAGAGATGCTGACCTATT
comH19 var 1-scr2	GCGATCAACTCCAACGACTAAT
comH19 var 1-scr3	GTGGCATCTACAGCGAAAGAGGT
comH19 var 1-scr4	GAGGCTATGGAGCGACCTAT
comH19 var 1-scr5	GATGGACGAGACGAGGGAGGAGT

### RAP to investigate lncH19-RNA interaction

The cells were harvested and lysed in a specific polysome lysis buffer (50 mM Tris-HCl pH 7.0, 10 mM EDTA, 1% SDS, 200U/ml RNase inhibitor, and protease inhibitor cocktail) on ice for 1.30 h. Cell debris was removed by centrifugation at 13.000 x *g* for 20 min at 4 °C and the supernatant, containing the protein lysate, was quantified as described before. For each reaction 500 µg of total proteins was resuspended in the hybridization buffer (50 mM Tris-HCl pH 7.0, 1 mM EDTA, 1% SDS, 750 mM NaCl, 15% Formamide added extemporaneously) and incubated with 100 picomoles of biotin-labeled H19 probes or control probes (designed from Sigma-Aldrich) 4 h under moderate agitation on a tube rotator at room temperature. After three washes with a specific washing buffer (0.5% SDS, 2X SSC) the samples were incubated with 50 µl streptavidin-magnetic beads overnight on a tube rotator at room temperature. After three washes with the washing buffer, the samples were incubated with proteinase-K (20 mg/ml, cat. n° 1409006, Invitrogen) for 45 min at 50 °C and 10 min at 95 °C. Finally, the RNA complexes bound to the beads were purified using a commercially available miRNA purification Kit (NucleoSpin™ miRNA kit, cat. n° 740971.250, Macherey–Nagel), following the manufacturer’s instructions and analyzed through PCR and qRT-PCR.

### RNA immunoprecipitation (RIP)

The cells were processed and lysed by adding a specific polysome lysis buffer (25 mM Tris-HCl pH 7.4, 100 mM KCl, 0.5% Nonidet P-40, 5 mM DTT, 5% 40 U/ml RNase inhibitor and protease inhibitor cocktail) for 30 min on ice. The magnetic Protein G-beads were pre-incubated with 2 µg of antibody against RBFOX2 or hnRNPM (respectively cat. n° sc-271407, sc-20002 both from Santa Cruz Biotechnology) or specific control mouse IgG (cat. n° 62-6520 EMD Millipore Corp., USA) at room temperature. After 30 min the corresponding cell lysates were added, and the samples were incubated overnight at 4 °C. The samples were subjected subsequently to RNA purification and protein extraction. The RIP efficiency was verified by Western blot assay and the enrichment of specific mRNAs was verified by qRT-PCR assay.

### RNA sequencing

Next-generation sequencing experiments were performed by Genomix4life S.R.L. (Baronissi, Salerno, Italy). Indexed libraries were prepared with TruSeq Stranded totalRNA gold Sample Prep Kit (Illumina) according to the manufacturer’s instructions. Libraries were quantified using the TapeStation 4200 (Agilent Technologies) and Qubit fluorometer (Invitrogen Co.), then pooled such that each index-tagged sample was present in equimolar amounts, with a final concentration of the pooled samples of 2 nM. The pooled samples were subject to cluster generation and sequencing using an Illumina NextSeq550 System (Illumina) in a 2 × 75 paired-end format. The raw sequence files generated (.fastq files) underwent quality control analysis using FastQC (http://www.bioinformatics.babraham.ac.uk/projects/fastqc).

### Bioinformatic analyses

#### Data preprocessing

Removal of adapters, poly(A) sequences, low-quality nucleotides, and trimming with Cutadapt tool version 2.5 [19], alignment of reads to the reference genome using STAR version 2.7.5c [20], with the standard parameters for paired reads. The reference track was assembly Human obtained from GenCode (HG38 - Release 37 (GRCh38.p13)) [https://www.gencodegenes.org/human/].

#### Peak calling

The bioinformatics tool MACS2 [21] was used to predict the binding sites. This tool identifies statistically significantly enriched genomic regions using the MACS2 algorithm (Model-Based Analysis). Bedtools utilities have been used to annotate and characterize the peaks as located in exons or introns of protein-coding, non-coding RNA, and Pseudogenes transcripts.

#### Prediction of lncRNA-RNA interaction

The RIblast method has been used for the computational prediction of lncRNA–RNA interactions [22]. This method consists of two steps. First, the “database construction” step generates RIblast database files from FASTA formatted RNA sequences file. To this scope, we downloaded all the available to date FASTA formatted RNA unspliced sequences files from Biomart. Second, the “RNA interaction search” step searches for RNA-RNA interaction of a query sequence in the previously computed RIblast database. In this case, we used as a query the lncH19 sequence (i.e. ENST00000428066 transcript). The output is a prediction file providing the following information: Id, Query name, Query Length, Target name, Target Length, Accessibility Energy, Hybridization Energy, Interaction Energy, and BasePair. The overlapping predictions for each gene were grouped, assigning to the union of them the lowest value of the Interaction Energy.

The lncH19-RNA computationally predicted interactions have been used to filter the peaks experimentally obtained with the analysis of RNA sequencing data from lncH19 RAP, maintaining only those falling into the predicted binding site with an interaction energy lower than − 10. The genes involved in interaction with Interaction Energy lower than − 10 have been saved for further processing.

#### Binding Affinity prediction

The CatRAPID algorithm [23] has been employed to predict the binding affinity of RBPs (hnRNPM, RBFOX2, and SRSF1) and lncH19. This algorithm leverages secondary structure predictions in conjunction with hydrogen bonding and van der Waals calculations to estimate the binding affinity of protein-RNA pairs. Interaction propensity is a metric that gauges the probability of interaction between one protein and lncH19 based on the observed tendency of the components of ribonucleoprotein complexes to exhibit specific properties of their physico-chemical profiles.

#### ENCODE analysis

To investigate the existence of a complex made by lncH19 and RBPs that regulate mRNA splicing, RBFOX2 and hnRNPM peaks (enriched genomic region) derived from enhanced CLIP (eCLIP) data have been collected from the ENCODE portal. The eCLIP peak co-occurrences across RBPs enable the discovery of novel co-interacting complexes. In particular, the ENCSR328LLU experiment used for this analysis belongs to a work that enabled the mapping of targets for 150 RBPs in HepG2, creating a unique resource of RBP interactomes profiled with a standardized methodology [24]. For each RBP, the bed files downloaded from ENCODE portal have been annotated and the genes associated with each peak have been identified. The gene lists obtained from the filtered peaks of lncH19 and SFs peaks obtained from ENCODE have been intersected and represented with the Venn diagram.

### Immunofluorescence assay

Cells were fixed in 4% paraformaldehyde, permeabilized with 0.1% Triton X-100, and stained for 1 h at room temperature with RAC1b primary antibody (1:100, cat. n° 09-271 EMD Millipore Corp., USA). The secondary antibody used was DyLight 488 (1:500 cat n° 35552 Thermo Fisher Scientific). Samples were counterstained with Hoechst 3342 (1:1000, cat n° H3570 Life Technologies) for 30 min at room temperature to detect nuclei and analyzed by confocal microscopy (Nikon A1) median nuclear planes. Quantification of the RAC1B signal has been performed using NIS-Elements software. We used a confocal microscope to capture the nuclear plane of each field. Using software, we identified the nuclei as Regions of Interest (ROI) based on the Hoechst signal. The software then calculated the intensity of the signal in the green channel (RAC1B) within each ROI, providing a measure of the mean fluorescence intensity (MFI) relative to the nuclear region. We analyzed three different fields per condition. The experiments have been performed in triplicate.

### Western blot assay

SDS-PAGE and Western blotting were performed according to standard protocols. To obtain total protein extracts, SW620 and HCT116 cells were lysed using a lysis buffer (15 mM Tris/HCl pH 7.5, 120 mM NaCl, 25 mM KCl, 1 mM EDTA, 0.5% Triton X100, and protease inhibitor cocktail) for 1.30 h on ice. Cell debris was removed by centrifugation at 14,000 x *g* for 15 min at 4 °C and the supernatant, containing the protein lysate, was quantified through the Bradford assay method (Pierce™ Coomassie Plus Assay Kit, cat. n° 23236, Thermo Fisher Scientific, United States) using bovine serum albumin (BSA, cat. n° A2153, Sigma-Aldrich, United States) as a standard.

To obtain nuclear and cytoplasmic protein fraction was used the Nuclear Extract Kit (Active Motif, Belgium) following the manufacturer’s instructions. Briefly, cells were first washed with PBS/phosphatase inhibitors, the supernatant aspirated, and fresh ice-cold PBS/phosphatase inhibitors added. The cells were removed by gently scraping and the cell suspension was centrifuged for 5 min at 200 x *g* at 4 °C. Then the supernatant was discarded, and the cell pellets were resuspended in 1X hypotonic buffer and incubated 15 min on ice. After this incubation specific detergent was added and the samples were vortexed 10 s at the highest setting. After centrifugation for 30 s at 14,000 x g at 4 ºC, the supernatant (cytoplasmic fraction) was transferred into a pre-chilled microcentrifuge tube and stored at − 80ºC until ready to use. The pellet was resuspended in 50 µl Complete Lysis Buffer by pipetting up and down and incubated for 30 min on ice on a rocking platform set at 150 rpm. After 30 s of vortex at the highest setting the samples were centrifuged for 10 min at 14,000 x g at 4ºC. Finally, the supernatant (nuclear fraction) was transferred into a pre-chilled microcentrifuge and stored at − 80ºC for the following experiments.

A total of 20 µg of proteins obtained from standard cell lysates or from nuclear and cytoplasmic fraction, or the specific amount of protein samples derived from RIP or RNA pull-down assay (see the specific section in material and methods paragraph), was separated using Bolt Bis-Tris gel 4–12% (cat. n° NP0326BOX, ThermoFisher Scientific) and transferred on nitrocellulose membranes (cat. n° 1060000, Amersham™ PROTRAN™ GE Healthcare; Chicago, IL, USA). The correctness of loading samples was evaluated by the staining of the membranes with 0.1% red Ponceau in 5% acetic acid. The membranes were then blocked for 1.30 h at 4 °C in 5% BSA solution (5% BSA, 20 mM Tris, 140 mM NaCl, 0.1% Tween-20) and incubated overnight at 4 °C with the following primary antibodies: anti-RAC1b (1:1000, cat. n° 09-271 EMD Millipore Corp., USA), anti-hnRNPM1-M4 (1:500, cat. n° sc-20002, Santa Cruz Biotechnology), anti-RBM9/rbFOX2 (1:1000, cat. n° A300-864 A Bethyl Fortis, Montgomery, Texas), anti-SRSF1 (1:500, cat. n° 324500, Invitrogen), anti-b-actin and anti-Laminin B1(both from Santa Cruz Biotechnology 1:1000, respectively cat. n° sc-81178, sc-365962). After washing with Tris-buffered saline-Tween-20 (TBS-T, 20 mM Tris, 140 mM NaCl, 0.1% Tween-20) three times at room temperature, the membranes were incubated for 1 h at 4 °C with appropriate secondary antibody HRP-conjugated goat anti-rabbit IgG (1:10.000, cat. n° 31460, Invitrogen™, Thermo Fisher^®^ Scientific, United States) and anti-mouse IgG (1:10.000, cat. n° 7076, Cell Signaling Technology, United States). The chemiluminescent signal was revealed using a chemiluminescence solution (ECL™ Prime Western Blotting System, cat. n° GERPN2232, Cytiva, Germany) and detected using the ChemiDoc acquisition instrument (Bio-Rad, United States). The obtained images were analyzed with the Image Lab software (Bio-Rad).

### Cell cycle analysis

SW620 and HCT116 cells were seeded respectively at 3 × 10^4^ or 2.5 × 10^4^ per cm^2^. After 24 h, cells were transfected with H19-pFLAG-CMV-2 expression vector or empty pFLAG-CMV-2 as Negative Control. After 18 h of transfection, the cells were treated with 50µM of RAC1B inhibitor EHT-1864 (Santa Cruz Biotechnology, cat. n° sc-361175) for 4 h. Cells were harvested and washed twice in cold PBS and centrifuged. The pellet was carefully fixed in 70% cold ethanol while vortexing to avoid cell clumping and stored at -20 °C for at least 24 h. Cells were then pelleted and washed with cold PBS and resuspended in 1 ml of cold cell cycle buffer (30 µg/mL propidium iodide, Sigma-Aldrich; 100 µg/mL DNase-free RNase A, Sigma-Aldrich; in PBS, pH7.4) [25]. Samples were incubated at least for 1 h at 4°C in the dark before acquisition. Acquisition was performed on a FACS Aria III (Becton-Dickinson, Milan, Italy) equipped with a 488 nm laser in the PE channel (585 nm), using DIVA v.8 software (BD). Cells were gated on the SSC/FCS dot-plot to exclude debris and further gated on the SSC-W/SSC-H and then on the FSC-W/FSC-H dot-plot to exclude doublets, and the gate was applied on the PE-A histogram. Data were analyzed and deconvoluted using ModFit LT v6.0 (Verity Software, MA, USA).

### Statistical analysis

Data are presented as mean ± standard deviation (SD) of independent biological replicates *n* ≥ 3. The normal data distribution was assessed by the Shapiro-Wilk test. When data followed a normal distribution, the statistical significance of differences was analyzed using one-sample t-test, to compare the mean to a hypothetical mean, or a two-tailed Student’s t-test to compare the group; otherwise, a non-parametric method (respectively Wilcoxon or Mann-Whitney test). A p-value ≤ 0.05 was considered significant. Pearson correlation analysis was used to correlate the expression of couples of genes in tumor samples expressing higher levels of lncH19 compared to normal counterparts (*n* = 14). Statistical analyses were performed using GraphPad Prism 10 software (GraphPad Software, United States). *p*-values were indicated in the graphs.

## Results

### The long non-coding RNA H19 interacts with mRNA precursors and splicing factors

Intending to analyze more closely the molecular mechanism through which lncH19 promotes CRC, we performed RNA Sequencing starting from lncH19 RNA Antisense Precipitation (RAP) in SW620. (Supplementary File A)

**Fig. 1:**
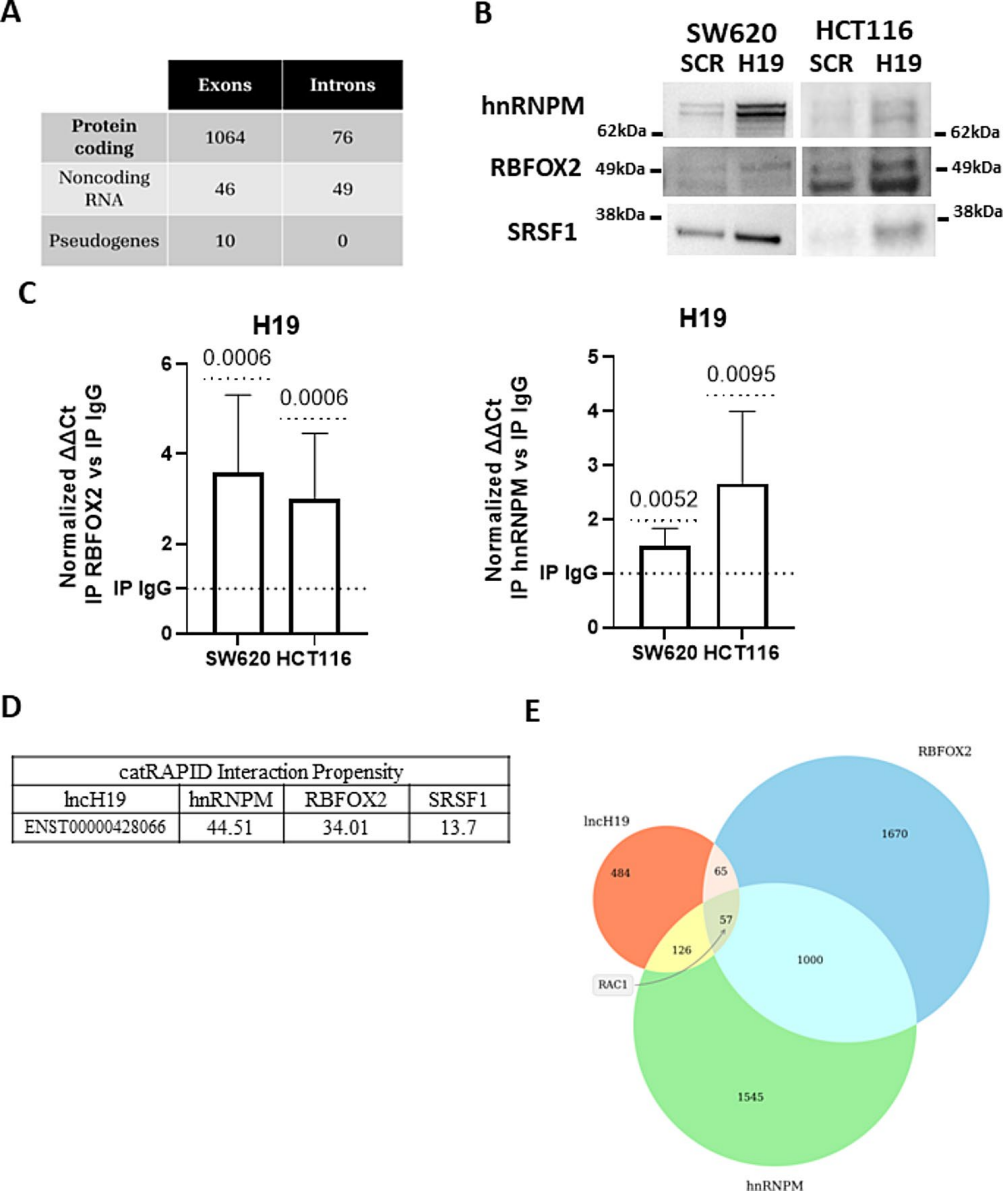
LncH19 interactors network. (**A**) Summary of sequences obtained by RNASeq from lncH19 Antisense Precipitation. (**B**) Western blot for the indicated proteins from lncH19 Antisense Precipitation in CRC cell lines (SW620 and HCT116). One representative experiment of three is shown. (**C**) RIP assay with anti-Fox2 (left) and anti-hnRNPM (right) antibodies to assess the binding of the RNABPs to lncH19 RNA in CRC cell lines (SW620 and HCT116); IgG was used as control. LncH19 levels were determined by qRT–PCR, normalized with input and presented as fold enrichment in RBFOX2 or hnRNPM relative to IgG. Statistical analyses were performed using normality test and t-test, p-value is shown in the graphs. (**D**) Binding affinity prediction between lncH19 and the indicated Splicing Factors by the use of catRAPID algorithm (**E**) Venn Diagram of lists of genes whose mRNA interacts with lncH19 (interactions are determined by the analysis of our RAP/RNA sequencing data and in-silico predictions), RBFOX2, and hnRNPM (interactions are determined by the analysis of eCLIP data from ENCODE portal

The table in Fig. 1A shows that, in CRC cells, the lncH19 binds a conspicuous number of mRNAs both in intron and exon sequences. This led us to hypothesize about the direct involvement of lncH19 in mRNA maturation and AS.

Several non-coding RNAs affect the AS process [15, 26, 27] by acting as natural antisense transcripts; these, interacting with pre-mRNAs by RNA–RNA base pairing can affect the selection of splice sites and the recruitment of alternative splicing factors (SFs) [26].

To validate our hypothesis, we investigated the binding between lncH19 and SFs involved in alternative splicing with pro-tumoral effects. Our study focused on RBFOX2 since it is among the most studied alternative splicing factors implicated in the development of EMT, cancer progression, and metastatic process [9, 10, 14, 28].

To investigate the binding between lncH19 and RBFOX2 we proceeded with lncH19 antisense precipitation (RAP) in two different CRC cell lines SW620 and HCT116. The western blot in Fig. 1B is the first evidence that, in colorectal cancer cell lines, lncH19 interacts with RBFOX2. We examined also the bond between lncH19 and RNABPs that are likely to be recruited by RBFOX2 to splicing sites, namely hnRNPM, SRSF1, and hnRNPC [13]. The western blot from lncH19-RAP, allowed us to detect the presence of hnRNPM and SRSF1, with the latter in smaller amounts than the former (Fig. 1B). However, no signal was detected for hnRNPC.

Moreover, by the use of the CatRAPID algorithm [23] we estimate the binding affinity between the identified splicing factors and lncH19. The results shown in Fig. 1D further confirm a higher binding affinity with hnRNPM and RBFOX2 and a lower affinity between with SRSF1.

The RNA Immune Precipitation (RIP) assays further confirmed the binding between the lncH19 and the RNABPs RBFOX2 and hnRNPM while no enrichment in lncH19 was obtained by SRSF1 immunoprecipitation (Fig. 1C and data not shown).

These data allow us to hypothesize the existence of a molecular platform that includes lncH19 and SFs. To identify putative targets of this complex, we have intersected the list of mRNAs bound by lncH19, as identified by the RAP analysis, with the RBP interactome for hnRNPM and RBFOX2 obtained from eCLIP profiling [24] (Fig. 1D).

The analyses identified 57 transcripts associated with lncH19 and containing binding sites for the investigated SFs, listed in Supplementary File 2.

Our investigation focused on RAC1, as it has been widely observed that its alternative splicing leads to RAC1B, a constitutive-active Ran GTPases which has been found to stimulate cancer cell proliferation, enhance epithelial-mesenchymal transition (EMT), and induce drug resistance [17].

### Both RBFOX2 and lncH19 control RAC1B expression in CRC cell lines

**Fig. 2:**
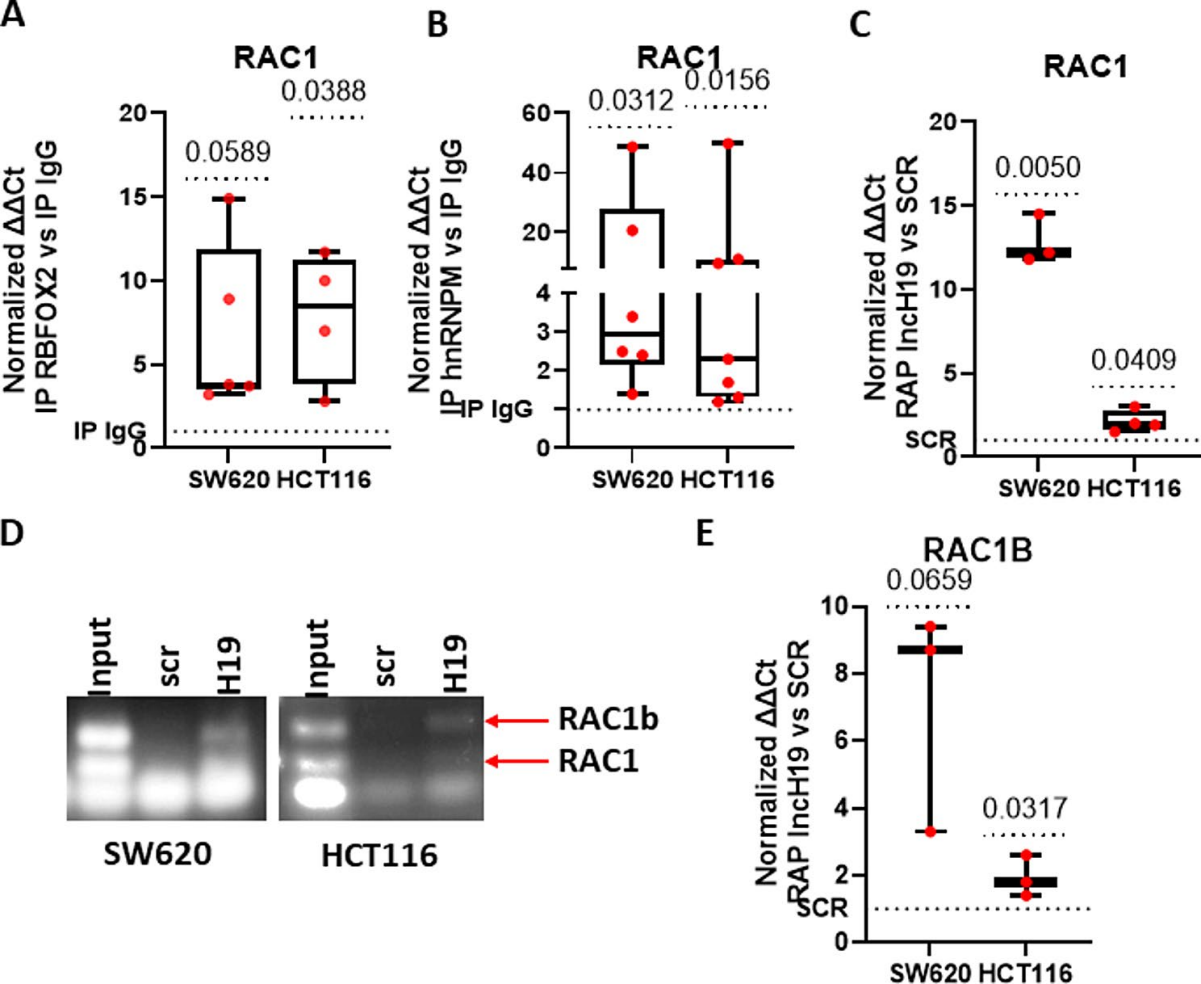
RBFOX2, hnRNPM and lncH19 bind RAC1 mRNA. (**A**-**B**) RIP assay with anti-Fox2 and anti-hnRNPM antibodies to assess the binding of the RNABPs to RAC1 RNA in HCT-116 and SW620 cells, IgG was used as control. RAC1 levels were determined by qRT–PCR normalized with input and presented as fold enrichment in RBFOX2 or hnRNPM relative to IgG. (Normality test and subsequent t-test (**A**) or Wilcox test (**B**). (**C**) RNA pull-down with biotin-labeled lncH19 oligonucleotides (lncH19 RAP) in CRC cell lines (SW620 and HCT116) to analyze the interaction between lncH19 and RAC1 mRNA. RAC1 levels were determined by qRT–PCR and presented as fold enrichment in lncH19 samples respect to RNA pull-down obtained with scrambled oligonucleotides (Normality test and t-test). (**D**) Agarose electrophoresis of splice-sensitive PCR RAC1-RAC1B from lncH19 RAP in CRC cell lines (SW620 and HCT116). One representative experiment of three is shown. (**E**) Quantitative analysis of RAC1B levels determined by qRT–PCR and presented as fold enrichment in lncH19 samples relative to input. Statistical analyses were performed using one sample t-test, the p-value is shown in the graphs

Firstly, by RIP for RBFOX2 (Fig. 2A) and hnRNPM (Fig. 2B), we confirmed that in CRC cells both the RNABPs bind RAC1 mRNA. Furthermore, RAP for lncH19, followed by real-time PCR, validated the data obtained by RNASeq, in particular the binding of the lncRNA to RAC1 (Fig. 2C). It’s important to note that the alternative RAC1 isoform, RAC1B, has an additional 57 nucleotides in exon 3b, resulting in the insertion of 19 new amino acids. Splice-sensitive PCR showed that lncH19 binds the longer isoform RAC1B (Fig. 2D), thus enforcing the hypothesis that the lncRNA is involved in AS, probably cooperated by RBFOX2. The binding was further investigated by real-time PCR (Fig. 2E).

Recent evidence correlated the activity of RBFOX2 in promoting EMT signature to RAC1B expression [29]. Moreover, it has been shown that RBFOX2 silencing but not hnRNPM silencing affects RAC1 splicing [13].

**Fig. 3:**
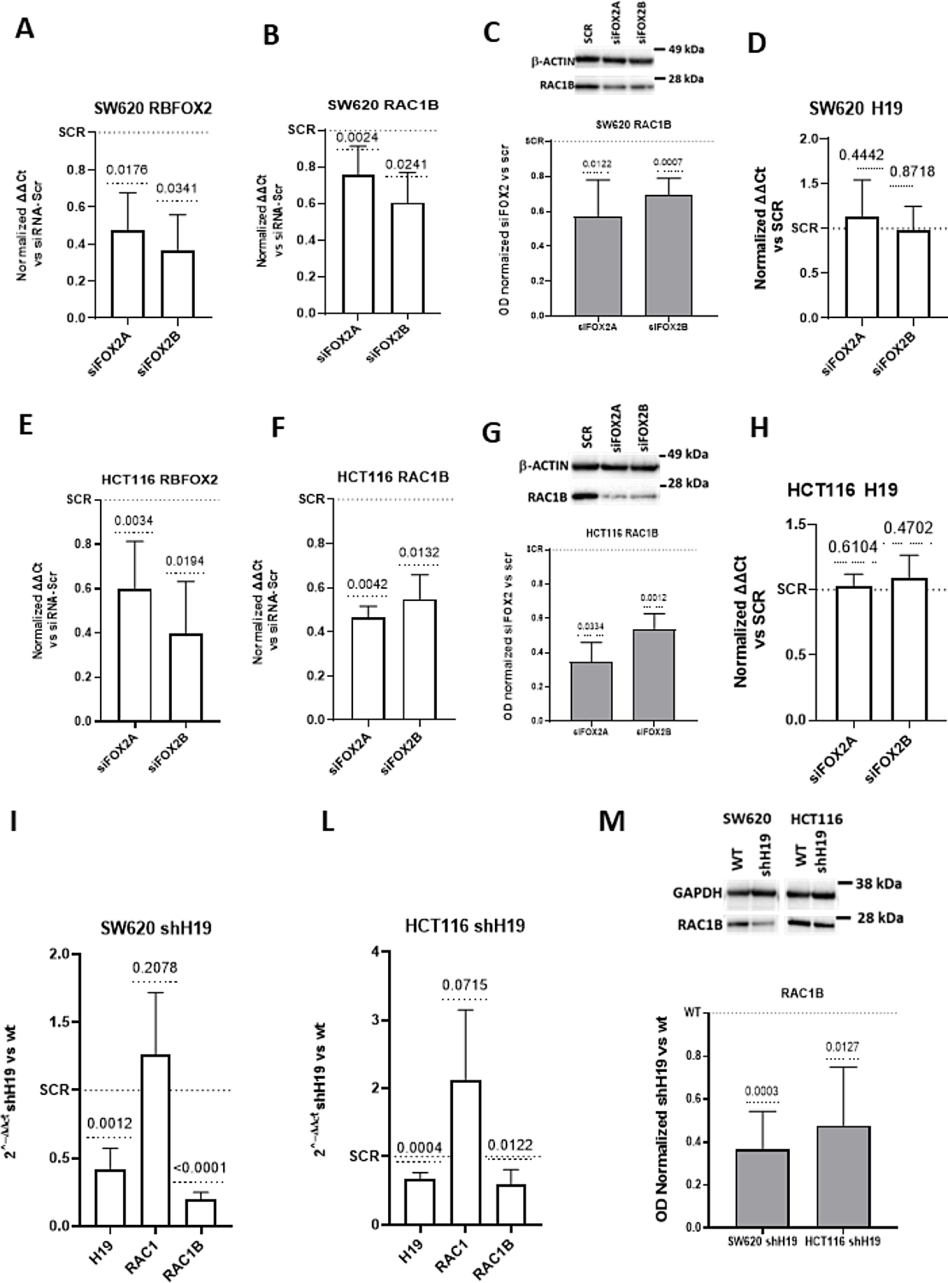
RBFOX2 is involved in RAC1 alternative splicing in CRC cells. (**A**-**B**-**D**, **E**-**F**-**H**) QRT-PCR for the indicated mRNA in CRC cells, silenced for RBFOX2 with two different siRNA. Graphs show 2-ΔΔct calculated in silenced cells respect to relative controls (Normality test and t-test). (**C**, **F**) Western Blot and densitometric analyses for RAC1B in SW620 and HCT116 silenced for RBFOX2 and relative controls. For densitometric analysis data are represented as normalized OD. (**I**-**L**) qRT-PCR of the indicated genes in SW620 and HCT116 silenced for H19, the graphs represent the 2^- ΔΔ ct of the indicated calculated respect the expression in control cells. (**M**) Western Blot of RAC1b protein levels in CRC cells (SW620 and HCT116) in H19 silenced cells and relative control cells. Statistical analyses were performed using normality test and t-test, p-value is shown in th e graphs

We transiently silenced two CRC cell lines for RBFOX2 (Fig. 3A, E). Transcriptional and protein data indicated that RBFOX2 silencing negatively affects RAC1B expression at transcriptional (Fig. 3B, F) and protein levels (Fig. 3C, G), thus confirming, also in CRC, the correlation between RBFOX2 and RAC1B expression. Notably, RBFOX2 silencing did not affect lncH19 expression (Fig. 3D, H).

Since the main aim of this study is to investigate if lncH19 takes part in RAC1 alternative splicing, we stably silenced CRC cell lines for the lncRNA (Fig. 3I-M).

Noteworthy, in H19-silenced cells RAC1B is significantly downregulated compared to wild-type cells both at transcriptional and protein level (Fig. 3I-M). The levels of RAC1 were not affected in lncH19-silenced cells enforcing the hypothesis that H19 plays a role in RAC1B splicing, rather than in RAC1 mRNA stabilization.

Neither RBFOX2 nor hnRNPM protein levels were altered by lncH19 silencing (Supplementary Fig. 3).

### LncH19 enhances oncogenes’ expression through RAC1B

**Fig. 4:**
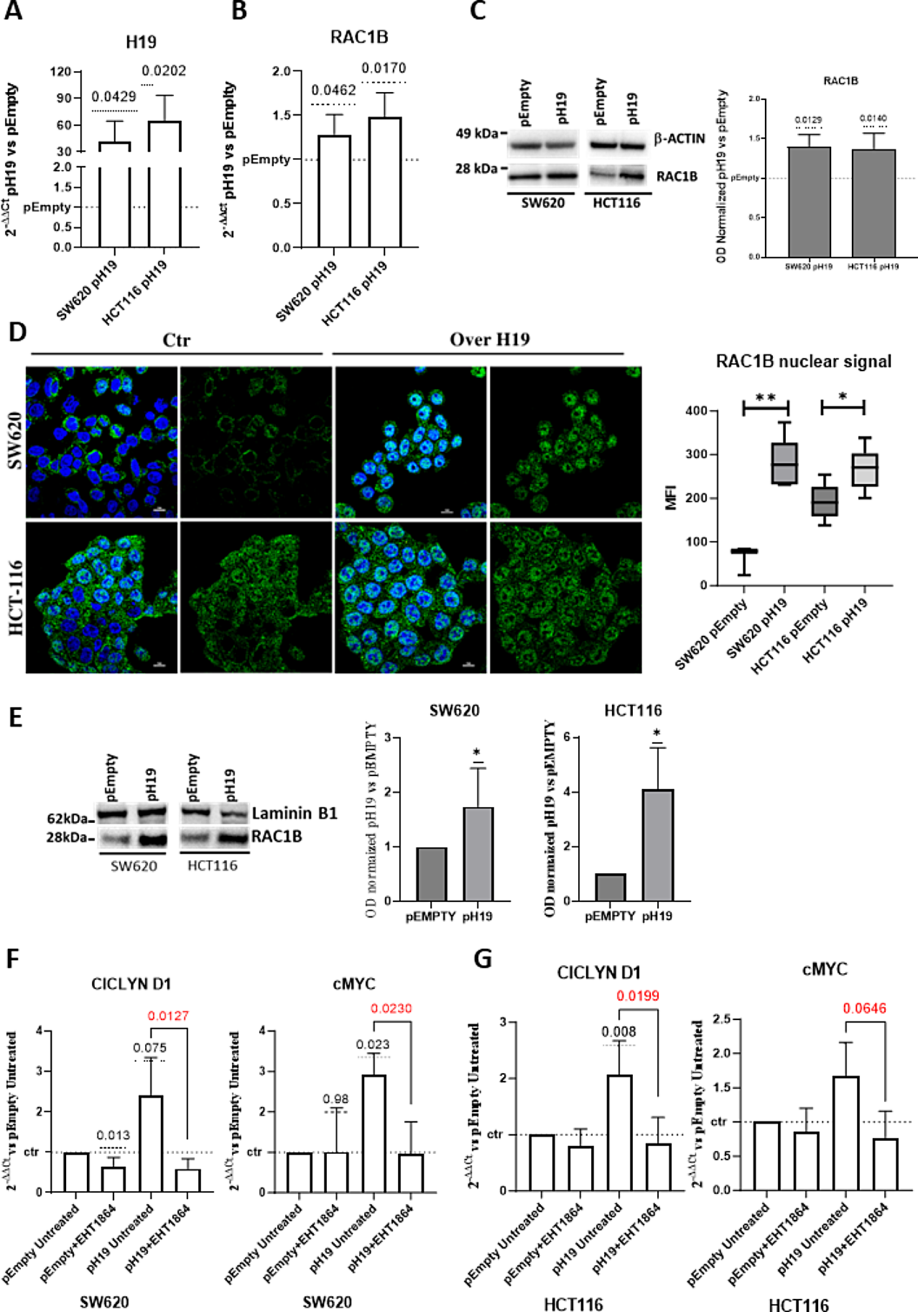
Overexpression of lncH19 upregulates RAC1B expression. (**A**) qRT-PCR to analyze H19 mRNA levels in CRC cells (SW620 and HCT116) transfected with lncH19 or empty vector. (**B**) qRT-PCR to analyze RAC1B mRNA levels in CRC cells (SW620 and HCT116) transfected with lncH19 or empty vector. (**C**) Western Blot for RAC1B in CRC cells (SW620 and HCT116), transfected with lncH19 or empty vector. lncH19 overexpression promotes RAC1B nuclear localization. (**D**) Representative confocal microscopy images of anti-RAC1b immunofluorescence showed nuclear localization of RAC1b in lncH19-overexpressing CRC cells, SW620 (upper panel) and HCT116 (lower panel). The analysis of the RAC1B nuclear signal is reported on the histogram. (**E**) Western blot analysis and densitometric analyses for RAC1B in nuclear protein fractions of CRC cells (SW620 and HCT116) overexpressing H19 cells and relative control cells. E-F) Overexpression of lncH19 upregulate CiclinD and c-Myc expression through RAC1B activity qRT-PCR for the indicated genes in SW620 (**F**) and HCT116 (**G**) transfected with pH19 or empty vector and treated or not with RAC1B inhibitor. Statistical analyses were performed using: normality test, one sample t-test to compare different conditions to pEmpty untreated cells, and Two-tails unpaired t-test to compare two groups *; p-value is shown in the graphs

We over-expressed lncH19 in CRC cell lines SW620 and HCT116 (Fig. 4A) to investigate its effects on RAC1B expression. Transcriptional and protein analyses revealed that lncH19 promotes RAC1B expression (Fig. 4B, C) moreover, immunofluorescence analyses for RAC1B showed a significant increase in the nuclear localization of the protein (Fig. 4D). Data confirmed by western blot from nuclear extracts (Fig. 4E).

In vivo and in vitro studies have demonstrated that RAC1B is overexpressed in CRC and high RAC1B expression correlates with high WNT activity and poor prognosis [18, 30, 31]. In particular, in CRC cell lines HCT116, it has been shown through chromatin immune precipitation that nuclear RAC1B, is recruited to the promoters of Wnt target genes c-Myc and Cyclin D1, acting as co-activator in B-catenin/TCF-mediated transcription enhancing the expression of the indicated genes [18, 30, 31].

Here we investigated if lncH19 may exert its oncogenic activity also through RAC1B-induced oncogenes’ activation.

To this aim, we treated H19 over-expressing cells with EHT-1864, the inhibitor of Rac-GTPase, able to inhibit also RAC1B activity [32]. Transcriptional data (Fig. 4F, G) confirmed that lncH19 over-expression promotes the expression of c-Myc and Cyclin D in both cell lines however, treatment with the RAC1B inhibitor leads to complete rescue. These data are supported by cell cycle analyses, which show a slight shift of the cell cycle in favor of the G2/M phase 24 h after lncH19 over-expression. Moreover, this shift is impeded by treatment with EHT-1864. (Supplementary Fig. 5)Overall, our data revealed a new axis, in the control of CRC gene expression passing through lncH19, RAC1B, and the Wnt target genes c-Myc and Cyclin D.

**Fig. 5:**
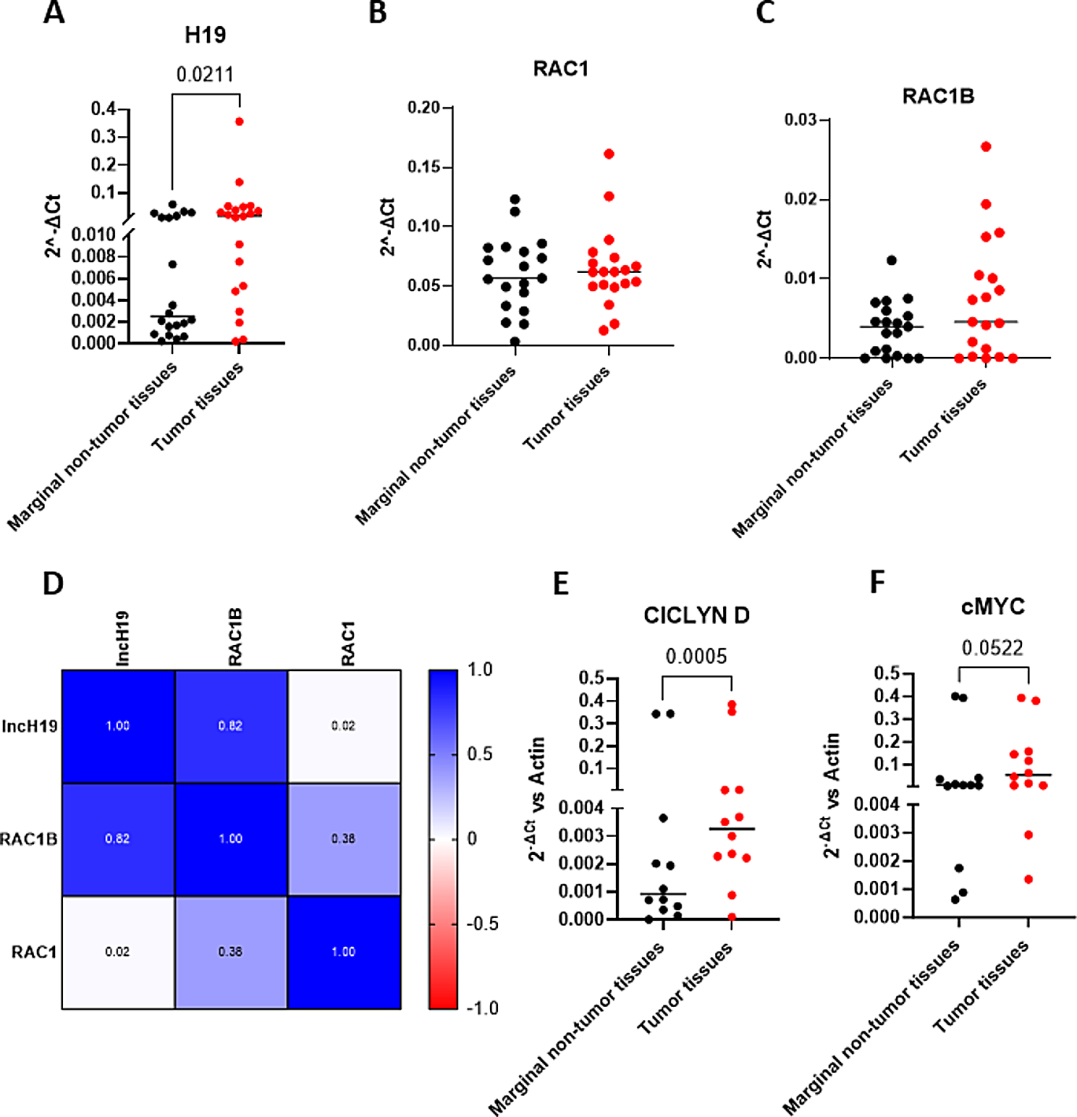
CRC tissues present higher levels of lncH19 compared to respective marginal non-tumor. (**A**-**C**) Gene expression levels for the indicated genes were examined by qRT-PCR in tumor and paired marginal non-tumor samples (*n* = 20). (**D**) Pearson correlation between lncH19, RAC1, and RAC1B expression analyzed in 14 colorectal cancer samples with lncH19 overexpressed compared to marginal non tumor tissue. (**E**-**F**) qRT-PCR for the indicated genes in colorectal cancer samples with lncH19 levels. All statistical analyses were performed using two-tail paired t-test, p-value is shown in the graphs

Finally, to validate this axis in vivo we investigated gene expression in biopsies from 20 patients with CRC. Real-time PCR (Fig. 5A) confirmed that CRC tissues present higher levels of lncH19 compared to respective marginal non-tumor tissue. No significative differences have been revealed in the comparison of RAC1 and RAC1B levels (Fig. 5B, C). However, by selecting the CRC samples with higher levels of lncH19 compared to their health counterpart (*n* = 14) Pearson Correlation analyses revealed a positive correlation between lncH19 and RAC1B (Fig. 5D). In addition, the same samples present higher levels of both oncogenes Cyclin D and c-Myc (Fig. 5E, F).

**Fig. 6:**
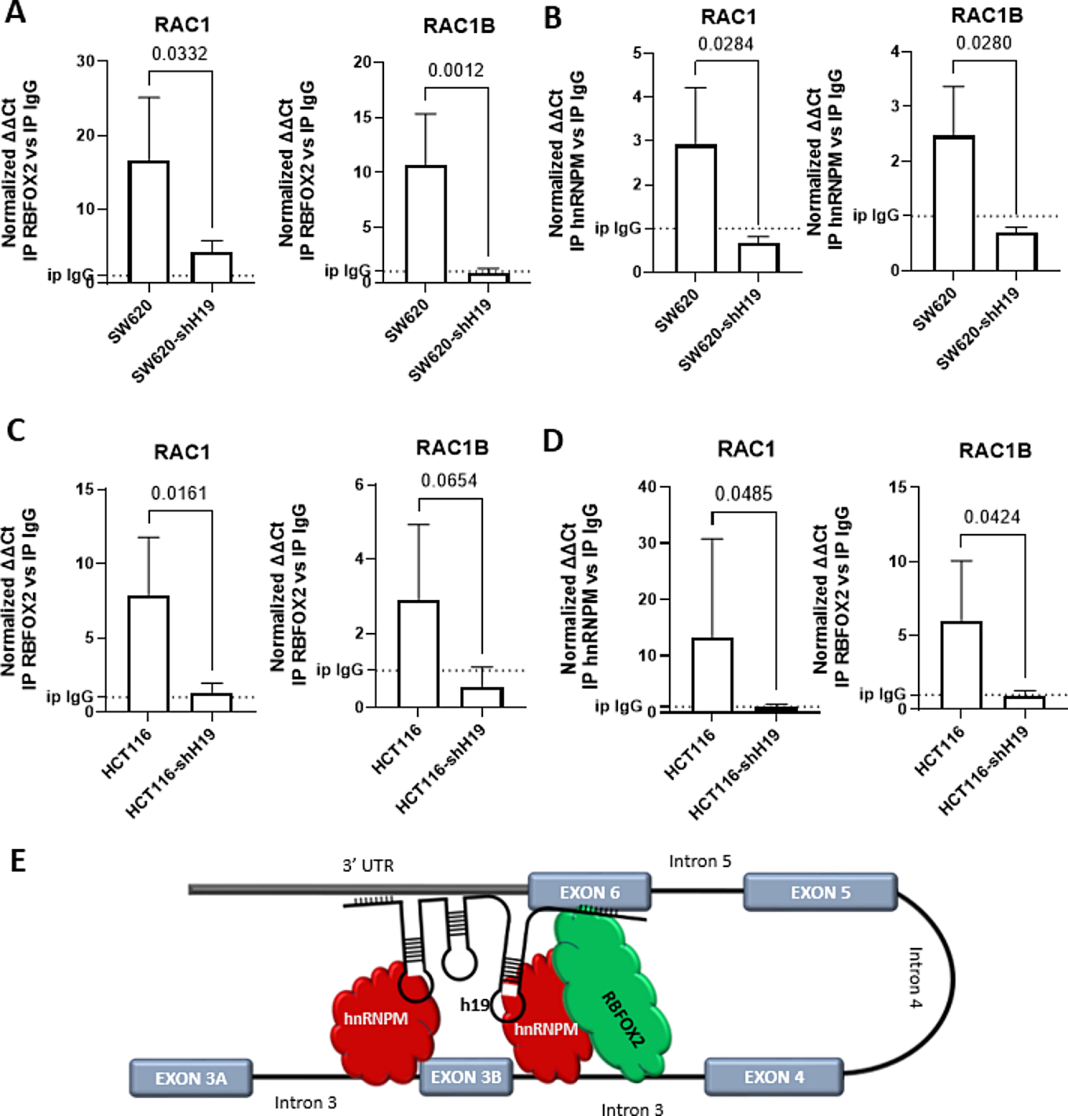
LncH19 is required to drive splicing factors on RAC1 mRNA. (**A**-**D**) QRT-PCR for RAC and RAC1b from RNA-immunoprecipitation (RIP) with anti-Fox2 (**A**, **C**) or anti-hnRNPM (**B**, **D**) antibodies in SW620 and HCT116 cells, RAC1 and RAC1B levels presented as fold enrichment in RBFOX2 or hnRNPM IP relative to IgG IP. Statistical analyses were performed using two-tail unpaired t-test to compare the binding between wt and shH19 silenced cells, p-value is shown in the graphs. (**E**) Schematic representation of the proposed model. Representation of binding sites position of the complex lncH19-hnRNPM-RBFOX2 with RAC1 unspliced mRNA

### LncH19 brings the splicing factors RBFOX2 and hnRNPM to RAC1 mRNA

The data here presented demonstrated that lncH19 directly affected RAC1 alternative splicing promoting exon 3B inclusion. To further investigate the molecular mechanism endorsing this process at first, we hypothesized that lncH19 might be functional for the assembly of the RNABPs (RBFOX2, hnRNPM, and SRSF1) in the splicing complex. However, co-IP revealed that lncH19 silencing did not affect the interaction between the SF (See supplementary 5).

We then hypothesized that lncH19 might have the role of bringing the splicing complex directly to RAC1 mRNA. RIP analyses confirmed this hypothesis. As shown in Fig. 6 the binding of RBFOX2 to RAC1 and RAC1B is strongly reduced in cells silenced for the lncH19 (Fig. 6A, C). The same concerns the binding of hnRNPM. (Fig. 6B, D).

## Discussion

The data here described revealed another piece of the complex mechanisms through which lncRNAH19 controls the tumor transformation process and the progression of CRC. Like for other lncRNAs, we demonstrated that lncH19 can work as a trans-acting element and control alternative splicing. Already in 2010 was described the retention in speckles of the lncRNA MALAT1 where it regulates AS by modulating the levels of active SR (serine/arginine-rich) proteins [33]. Other lncRNAs control AS in an indirect way, as for the linc01232; it has been shown to interact with the splicing factor hnRNPA2/B1 and stabilize it by preventing ubiquitination and degradation of hnRNPA2/B1 [34]. Based on both the experimental data and bioinformatic analyses, we hypothesize that lncH19 binds to the 3’UTR of RAC1 mRNA. Additionally, our findings suggest that lncH19 interacts with the SFs RBFOX2 and hnRNPM, bringing them to their respective binding sites that flank exon 3 A. As a result, lncH19 impedes the exclusion of exon 3 A (Fig. 6E).

In a study investigating alternative splicing of RAC1 in CRC, Gonçalves and colleagues [35] found that the expression of SRSF1 increases the retention of exon3A. Our research adds further insight into the mechanism that promotes the maturation of RAC1B. We found that SRSF1 is part of a splicing complex including hnRNPM, and RBFOX2 and demonstrated that the complex is specifically conveyed on RAC1 mRNA by lncH19. It appears from our experimental data that the lncRNA mainly binds RBFOX2 and hnRNPM however, the architecture of the lncH19-SF bond requires further definition.

The supposed correlation between lncH19 and RAC1B was confirmed in vivo where higher expression of the lncH19 in tumor samples was positively correlated with RAC1B expression.

It is to be noted that we identified 57 transcripts with both complementary regions for lncH19 and binding sites for the SFs RBFOX2 and hnRNPM (Supplementary Fig. 2). Although further studies are required to demonstrate this, there is no reason to doubt that the mechanism of action proposed here for RAC1 maturation can be extended to other transcripts. This would explain why lncH19 over-expression has such pleiotropic effects in CRC. Our next effort will be first to investigate among the 57 transcripts, those whose alternative splicing is involved in or associated with tumor progression.

It is still unclear whether the model identified in CRC can be applied to other types of tumors. However, we have developed a strategy to explore potential similarities and identify other targets in tumors that exhibit overexpression of this lncRNA.

The data here presented provided new insights into the mechanisms of action of lncH19, but also highlighted a previously unknown axis through which lncH19 exerts its pro-tumoral activity in CRC. We and others have already associated the expression of lncH19 with an enhanced Wnt pathway activation in CRC models. In particular, Ding and collaborators demonstrated that lncH19 enhances b-catenin target gene activation by sponging the miR-29b-3p [36]. In our study lncH19 intragenic miRNA (miR-675-5p), controlling Glycogen Synthase Kinase 3β activity, promotes b-catenin nuclear localization [37]. Moreover, it has also been demonstrated that lncH19 interacting with macroH2A histone variants regulates transcription of cyclin-dependent kinase 8, with a consequent effect on β-catenin activity [38]. Studies conducted in CRC have already shown [30] that RAC1B migrates into the nucleus where it associates with the β-catenin-TCF complex promoting the expression of c-Myc and Cyclin D. Here we demonstrated that through the maturation of RAC1B, lncH19 exerts control on the β-catenin, target genes. The over-expression of H19 leads to the transcriptional up-regulation of c-Myc and Cyclin D, however, the use of a RAC1 inhibitor is sufficient to bring the expression of these genes back to their basal level.

## Conclusions

The results here described unveiled a new molecular mechanism through which the lncH19 exerts its activity in tumor progression, by selecting which transcripts should undergo alternative splicing. This would explain the pleiotropic effects attributed to lncH19 over-expression in CRC and further pushes towards the possible use of antisense RNA to support anti-tumor therapy.

### Electronic supplementary material

Below is the link to the electronic supplementary material.

Supplementary Material 1

Supplementary Material 2

Supplementary Material 3

Supplementary Material 4

Supplementary Material 5

## Data Availability

The raw data supporting the conclusion of this article will be made available by the authors, without undue reservation.
