# Supplementary material for: Regulatory role of lncH19 in RAC1 alternative splicing: implication for RAC1B expression in colorectal cancer
Source: J Exp Clin Cancer Res. 2024 Aug 5;43:217. doi: 10.1186/s13046-024-03139-z (PMC11299361; doi:10.1186/s13046-024-03139-z)
Supplement: Supplementary file 3 — Supplementary Material 3 [file 13046_2024_3139_MOESM3_ESM.pptx]

## Slide 1
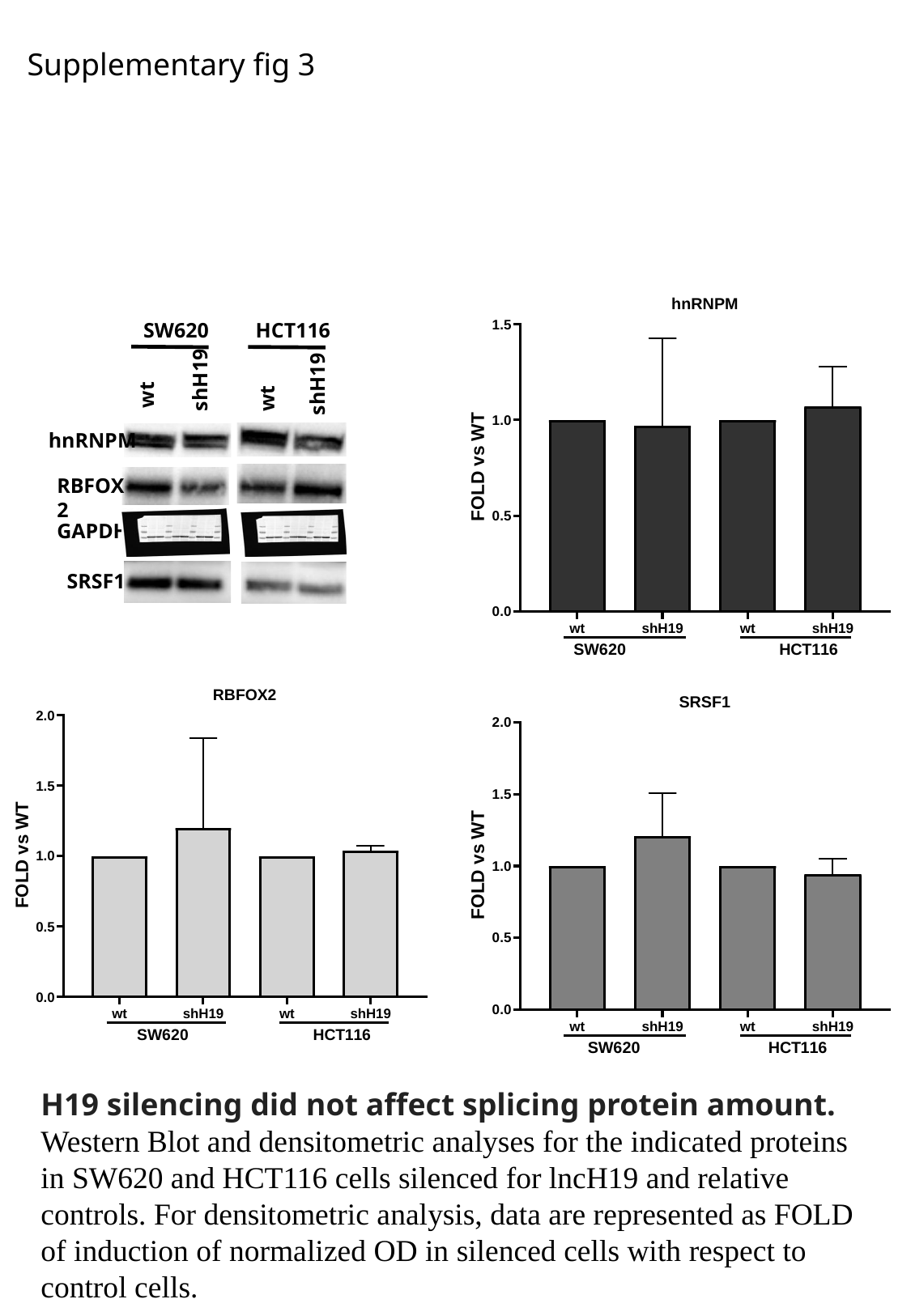

Supplementary fig 3
HCT116
SW620
shH19
shH19
wt
wt
GAPDH
SRSF1
hnRNPM
RBFOX2
H19 silencing did not affect splicing protein amount. Western Blot and densitometric analyses for the indicated proteins in SW620 and HCT116 cells silenced for lncH19 and relative controls. For densitometric analysis, data are represented as FOLD of induction of normalized OD in silenced cells with respect to control cells.

## Slide 2
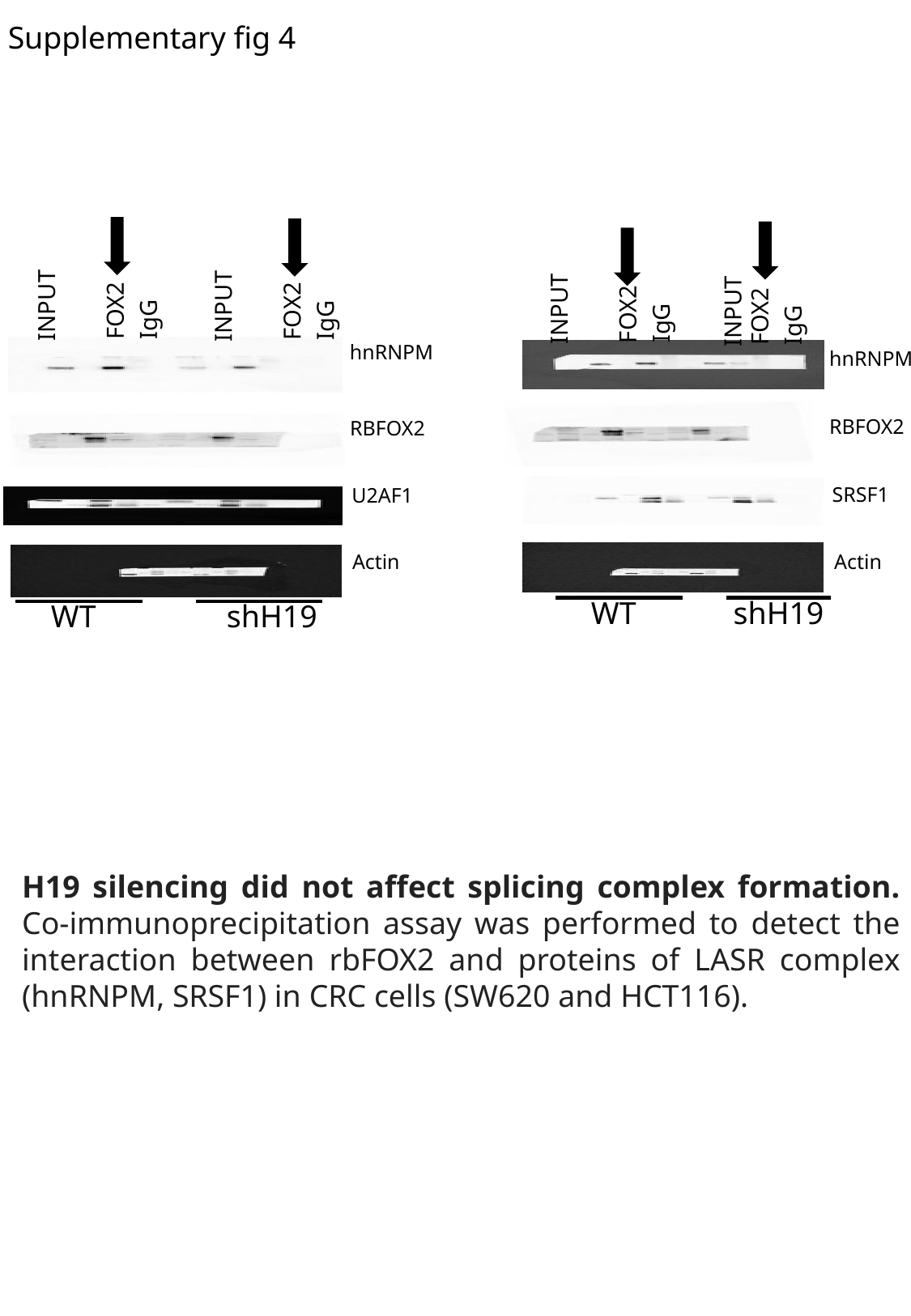

Supplementary fig 4
INPUT
INPUT
INPUT
FOX2
FOX2
INPUT
FOX2
FOX2
IgG
IgG
IgG
IgG
hnRNPM
hnRNPM
RBFOX2
RBFOX2
SRSF1
U2AF1
Actin
Actin
WT
shH19
WT
shH19
H19 silencing did not affect splicing complex formation. Co-immunoprecipitation assay was performed to detect the interaction between rbFOX2 and proteins of LASR complex (hnRNPM, SRSF1) in CRC cells (SW620 and HCT116).

## Slide 3
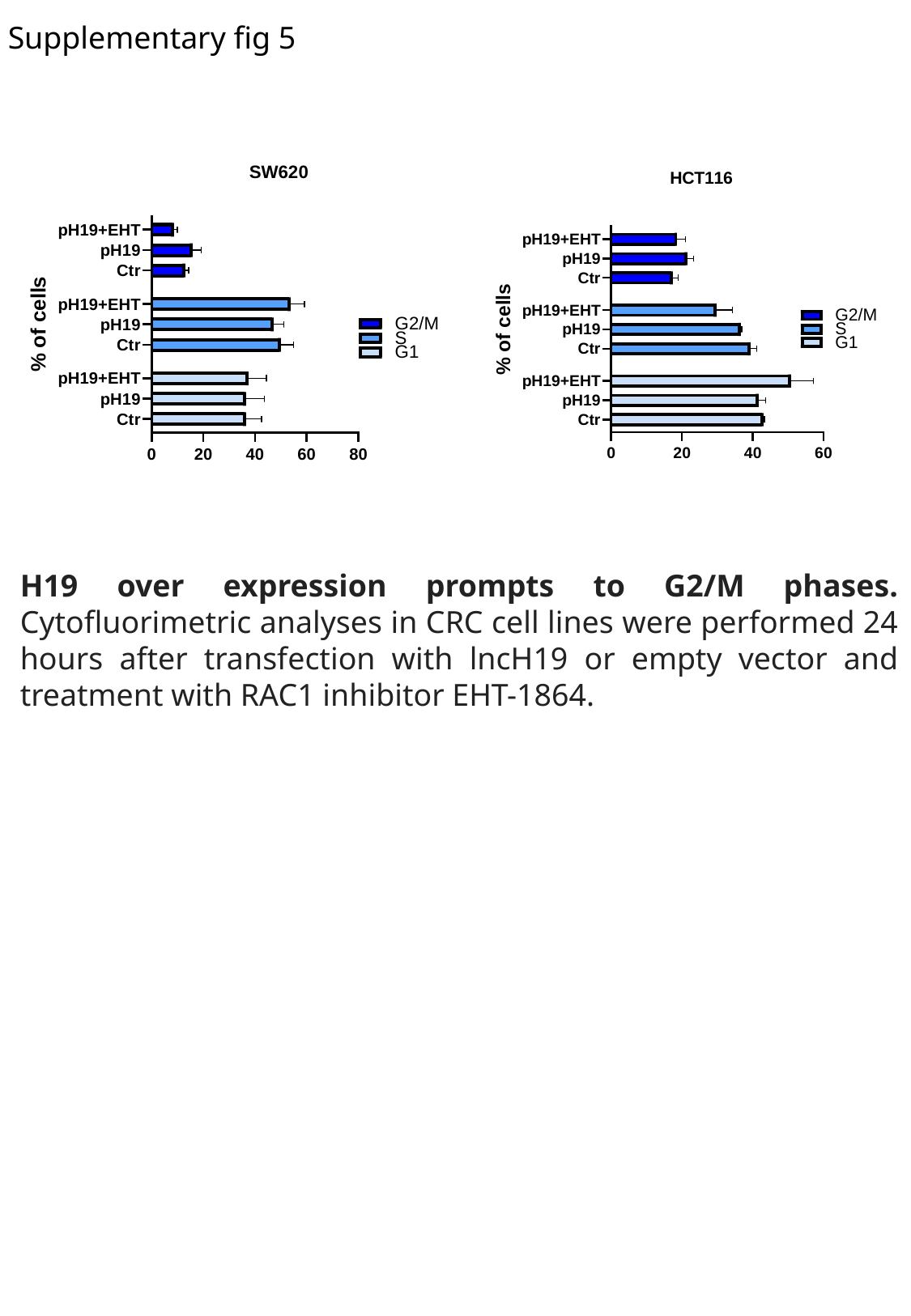

Supplementary fig 5
H19 over expression prompts to G2/M phases. Cytofluorimetric analyses in CRC cell lines were performed 24 hours after transfection with lncH19 or empty vector and treatment with RAC1 inhibitor EHT-1864.
