# Supplementary figures and images for: Regulatory role of lncH19 in RAC1 alternative splicing: implication for RAC1B expression in colorectal cancer
Source: J Exp Clin Cancer Res. 2024 Aug 5;43:217. doi: 10.1186/s13046-024-03139-z (PMC11299361; doi:10.1186/s13046-024-03139-z)

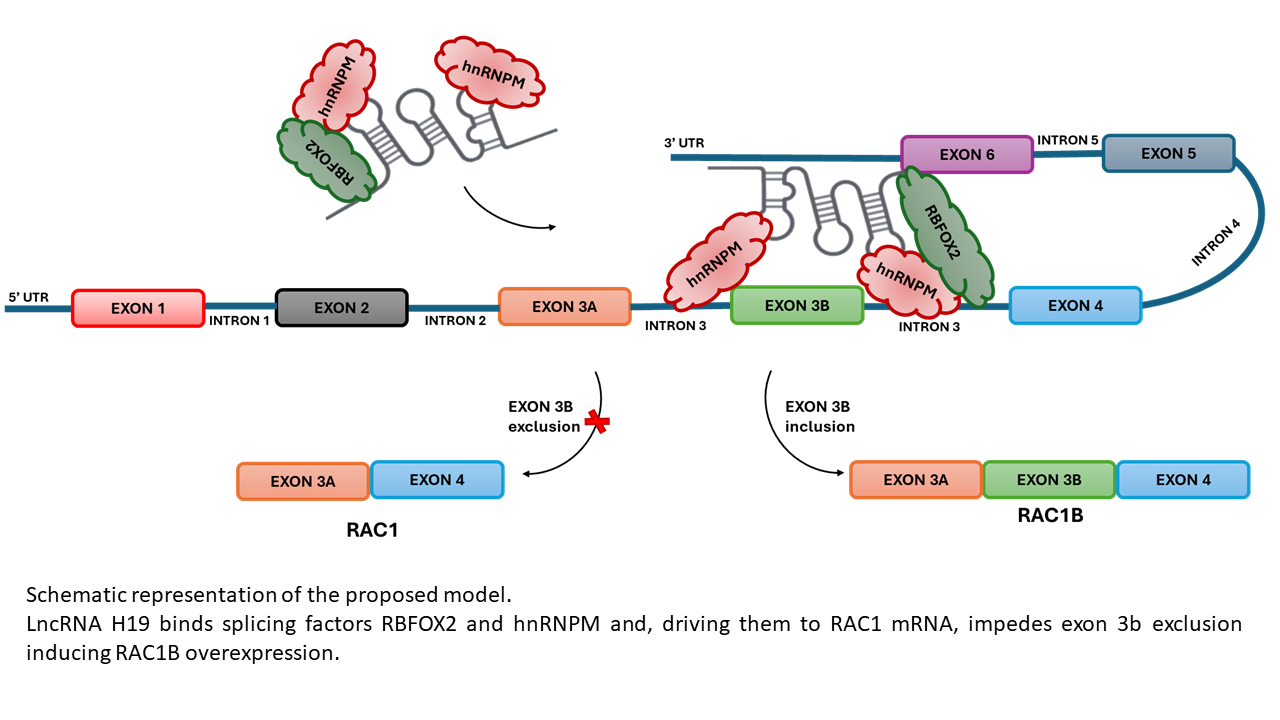

Supplement: Supplementary file 4 — Supplementary Material 4 [file 13046_2024_3139_MOESM4_ESM.tif]
