# Supplementary material for: Regulatory role of lncH19 in RAC1 alternative splicing: implication for RAC1B expression in colorectal cancer
Source: J Exp Clin Cancer Res. 2024 Aug 5;43:217. doi: 10.1186/s13046-024-03139-z (PMC11299361; doi:10.1186/s13046-024-03139-z)
Supplement: Supplementary file 5 — Supplementary Material 5 [file 13046_2024_3139_MOESM5_ESM.pdf]

Point by Point Response to Reviewers

Reviewer 1

Reviewer #1: General comments/Results
1. Did the authors also hypothesize a role for miRNA675-5p? 2. Have they tested the use of miRNA675-5p inhibitor in H19-induced effect? 4. Did the authors analyze miRNA675-5p expression in biopsies?

Firstly, we want to thank the reviewer for his/her interesting comments and suggestions. We strongly believe that miR-675 cooperates with the lncH19 in the progression of colorectal cancer. In our previous manuscripts, we demonstrated that miR-675-5p is over-expressed in colorectal cancer cell lines and tissues; moreover, we found that miR-675-5p enforces the pro-EMT activity of the lncH19 especially under hypoxic stimulation (https://doi.org/10.18632/oncotarget.14464).

Here we aimed to investigate a new mechanism of action for the lncH19 due to its ability to bind simultaneously RNA and proteins. We are interested in exploring whether miR-675-5p can affect the interaction of lncH19-splicing factors. However, further studies are required to examine these aspects. We hope to collect data on this for a future manuscript.

3. Did the authors consider also a non-metastatic line, such as SW480?

The reviewer's question brings up an interesting aspect. Although our data suggests that lncH19 binds RBFOX2 in SW480 cell line, we could only confirm this binding using RIP Anti RBFOX2 and could not efficiently proceed with the RNA pull-down assays. We suspect that the lower levels of H19 in SW480 cell line may be the reason for this, moreover, it would be in line with the lower aggressivity of SW480 cells compared to SW620 and HCT116 (recently the correlation between lncH19 levels and aggressive phenotype has been confirmed also in doi: 10.1186/s13046-020-01783-9). However, we don't have enough data to make a definitive conclusion on this matter. Below is the graph showing the baseline values of H19 in the three different cell lines.

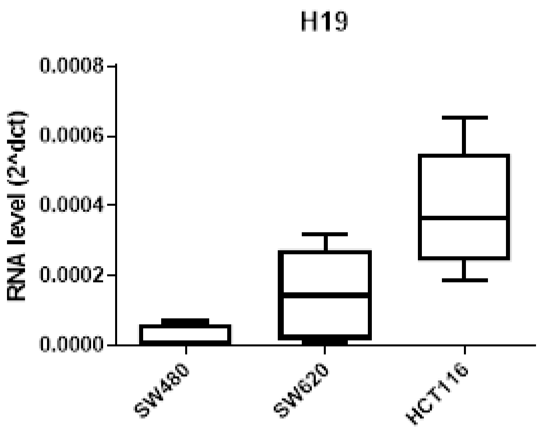

5. In 3.3 paragraph, the authors indicate cyclin D and c-myc as studied oncogenes. It is worth explaining why just those two oncogenes. Did the authors also study any oncosuppressor?

Thanks for this appropriate observation, which also allowed us to stress this aspect in the main text.

Bapat group in (https://doi.org/10.3892/ijo.2011.1066) demonstrated that the isoform RAC1B resides in the nucleus of cancer cells, including HCT116, and in particular by chromatin immune precipitation they demonstrated that RAC1B is located in the promoters of the c-Myc and Cyclin D1 working as co-activator in B-catenin/TCF-mediated transcription. Here we investigated c-Myc and Cyclin D1 expression only as indicators of the nuclear activity of RAC1B. This aspect has been further clarified in the text.

See results page 16:

In vivo and in vitro studies have demonstrated that RAC1B is overexpressed in CRC and high RAC1B expression correlates with high WNT activity and poor prognosis [18, 29, 30]. In particular, in CRC cell lines HCT116, it has been shown through chromatin immune precipitation that nuclear RAC1B, is recruited to the promoters of Wnt target genes c-Myc and Cyclin D1, acting as co-activator in B-catenin/TCF-mediated transcription enhancing the expression of the indicated genes [18, 29, 30].

6. Considering that involvement of cyclin D has been described, has the effect on the cycle (i.e. FACS) or at least the expression of the relative cdk been evaluated to get an idea of the actual functionality of the oncogene?

Following the reviewer's suggestion we investigated the cell cycle in our cellular models. The graph below shows that already 24 hours after lncH19 over-expression, a slight shift of the cell cycle in favour of the G2/M phase is observed, this is impeded by EHT drug treatment. This data has been presented in the revised manuscript as supplementary figure 5

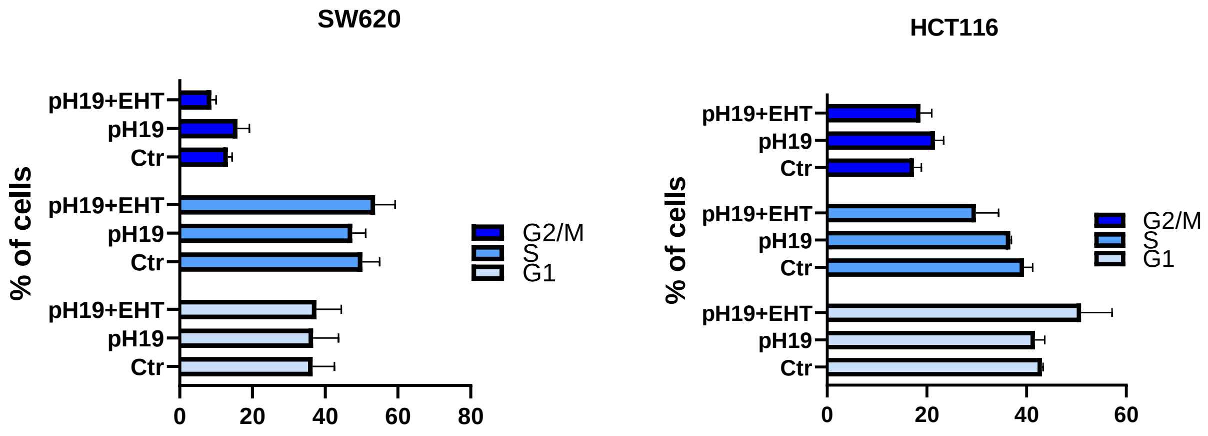

Figures

1. In figure 1C, right panel, it is indicated the IP for RBFOX2 ma I'm afraid it's wrong. I think that the graph refers to RNPM. 2. In figure 2C and 6A the name "IP IgG" is overlapped with the axis. please correct

We apologize for the errors and thank the reviewer for the meticulous review, the two graphs have been corrected.

Materials and methods

1.Are the authors sure that the beta-actin reverse sequence is correct? Please check on BLAST

We apologize for the careless copy and paste of the sequence, and we thank the reviewer for allowing us to revise this. Correct oligo-sequence has been added in the manuscript.

2. In the immunofluorescence assay paragraph, the authors should also explain the image analysis method. kindly add

Thanks for bringing attention to this deficit.

This method has been added to the text:

Quantification of the RAC1B signal has been performed using NIS-Elements software. We used a confocal microscope to capture the nuclear plane of each field. Using software, we identified the nuclei as Regions of Interest (ROI) based on the Hoechst signal. The software then calculated the intensity of the signal in the green channel (RAC1b) within each ROI, providing a measure of the mean fluorescence intensity (MFI) relative to the nuclear region. We analysed three different fields per condition. The experiments have been performed in triplicate.

We appreciate the reviewer's effort in revising our work. This feedback helped us identify and address previously unnoticed shortcomings.

Reviewer #2: The manuscript entitled "Regulatory role of lncH19 in RAC1 alternative splicing: implication for RAC1B

expression in colorectal cancer" focuses into the function of the lncRNA H19 as an oncogene in colorectal cancer (CRC) by modulating alternative splicing. The authors demonstrate that in CRC cells (SW620 and HCT116), lncH19 interacts with splicing factors (SFs) RBFOX2 and hnRNPM. In addition thay find that both lncH19, RBFOX2, and hnRNPM bind the GTP-ase RAC1, whose alternatively spliced isoform, RAC1B, has various roles in malignant transformation. In particular, they show that lncH19 preferentially binds to RAC1B. Loss and gain of expression experiments indicate that lncH19 is required for RAC1B expression and, via RAC1B, induces an increase in c-Myc and Cyclin-D. Statistical analyses on in vivo experiments using biopsies from CRC patients reveal a positive correlation between lncH19 and RAC1B. These samples also showed higher expression levels of cMyc and CiclynD. Finally, the authors demonstrated that lncH19 drives the splicing factors RBFOX2 and hnRNPM to RAC1, allowing exon retention and RAC1B expression. The manuscript is well written and structured, and the topic is of significant interest.

We want to thank the reviewer for his/her efforts in reviewing the manuscript, and we are happy that the reviewer found our manuscript well-written and the topic of significant interest.

We greatly appreciate the attention given by the reviewer to the different parts of the work and especially the constructive comments that helped us clarify some aspects of the manuscript that had been overlooked in hindsight.

* The authors conducted their experiments using the HCT116 and SW620 cell lines. It would be helpful for readers if the authors could provide a clearer rationale for selecting these specific cell lines.

We thank the reviewer for this observation, in the beginning, we performed our preliminary experiments by proceeding with 3 CRC cell lines routinely used in our laboratory (SW480-SW620-HCT-116). Our data suggests that lncH19 binds RBFOX2 also in SW480 cell line, we confirmed this binding by Anti RBFOX2 RIP but we were unable to efficiently proceed with the RNA pull-down assays. We suspect that the lower levels of H19 in the SW480 cell line may be the reason for this (see the graph below).

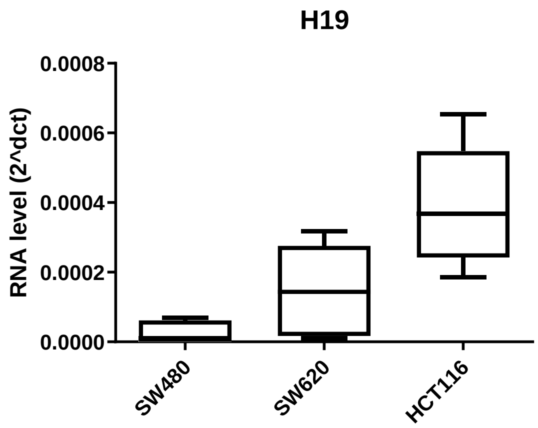

The lower level of lncH19 in SW480 cells is in line with the less aggressive phenotype of these cells compared to both HCT116 and SW620 and with the data recently published in doi: 10.1186/s13046-020-01783-9. However, we don't have enough data to make a definitive conclusion on this matter.

For this reason, we decided to proceed with the CRC cell lines SW620 and HCT-116.

* The authors illustrate the interaction between LncH19 and RBFOX2 (FIG.1B). Clarification on why the RBFOX2 splicing factor was chosen would enhance understanding.

-Concerning the choice of RBFOX2 we described in the introduction the data from the literature that attributed to RBFOX2 a role as an inductor of oncogenic splice-switching that drives an invasive phenotype in cancer (please see references 9-14), following the reviewerìs suggestion this point as been further stressed in the introduction.

RBFOX2 is the master regulator of tissue-specific alternative splicing, implicated in the development of ovarian and breast cancer as well as in EMT [9-11]. RBFOX2 recognizes different sets of alternatively spliced RNAs, sometimes also without a specific binding motif thanks to its interaction

with multiple proteins [12, 13]. More recently it has been demonstrated its implication in the microexon splicing, associated with CRC metastasis [14]. Overall, the data in the literature attributed to RBFOX2 a role as an inductor of oncogenic splice-switching that drives an invasive phenotype in cancer.

Additionally, quantifying the binding affinity between the investigated splicing factors (RBFOX2, hnRNPM, and SRSF1) and LncH19 could provide valuable insights.

We thank the reviewer for this interesting suggestion. The CatRAPID algorithm (DOI: 10.1093/nar/gkab393) was used to predict the binding affinity of the RBPs and lncH19. The results obtained are shown in the graph below. This algorithm leverages secondary structure predictions in conjunction with hydrogen bonding and van der Waals calculations to estimate the binding affinity of protein-RNA pairs. The table below presents the values of interaction propensity for hnRNPM, RBFOX2, and SRSF1. The results indicate a higher binding affinity of lncH19 with hnRNPM and RBFOX2 and a lower affinity with SRSF1.

Interaction propensity between RBP and lncH19 predicted by catRAPID.

	catRAPID Interaction Propensity		
lncH19	hnRNPM	RBFOX2	SRSF1
ENST00000428066	44.51	34.01	13.7

This data has been added to the results, the strategy used for bioinformatic prediction has been described in material and methods, and the table has been included in Figure 1.

Moreover, a higher quality immunoprecipitation for the SW620 cell line would strengthen the experimental findings.

-The Western blot from the immunoprecipitation has been improved with a higher-quality experiment (figure 1 B).

* The authors confirmed that the splicing factor RBFOX2 binds to RAC1 mRNA (FIG2A). However, the qPCR results for the SW620 cell line show no statistical significance ($p = 0.0702$). Additionally, in the HCT116 cell line (FIG2B), the standard deviation is notably high. The authors should provide clarification on how they achieved statistical significance despite the high variability in the data.

We appreciate the reviewerìs feedback and would like to take this opportunity to explain our perspective.

The data presented in Figure 2A-B demonstrate the binding between the SFs (RBFOX2 and hnRNPM) and the lncRNAH19. We consistently observed a specific binding, since the RNA analyses from SFs pull-down always led to an enrichment of the lncH19 compared to the IgG pulldown. However, the level of enrichment varied because of experimental variability beyond our control. To address this, we replicated the experiments multiple times and included all the biological replicates in the graph, understanding that this would increase the standard deviation.

We noticed that the previous graph did not effectively show the importance of the test or the number of replicates. Therefore, in the new Figure 2, we replaced the column graph with a box plot that

displays all the replicates and the minimum and maximum values obtained. We think the new graph better illustrates the consistency of lncH19 binding to SFs in all the experiments.

In response to the concerns regarding Figure 2A, we conducted an additional replicate which reduced the p-value to 0.058.

Regarding the statistics for Figure 2B as described in the manuscript, we first tested the normal distribution of the data using the Shapiro-Wilk test. If the sample had a normal distribution, we used a one-sample t-test to compare the mean to a hypothetical mean. If the data did not have a normal distribution, we used the non-parametric Wilcoxon test. Since the data in Figure 2B did not have a normal distribution, we used the Wilcoxon test, and the result is reported in the graph.

* The evidence that LNCH19 binds to RAC1B more than RAC1, as shown by real-time PCR (FIG2E), is not statistically significant for the SW620 cell line.

We acknowledge the reviewer's feedback. The data presented in Fig. 2D-E is not intended to show that lncH19 binds to RAC1B more than RAC1. Rather, it provides evidence that lncH19 can bind to the longer isoform of RAC1. This data led us to hypothesize that lncH19 may interact with RAC1 mRNA before its maturation and be involved in alternative splicing. We have revised the wording in the text to make this aspect clearer to the reader.

* The authors observed variations in RAC1b expression upon transient silencing of RBFOX2. It would be beneficial if the authors provided details in the Materials and Methods section on how they achieved transient silencing of RBFOX2

We apologize for the serious forgetfulness, the material and method used to silence RBFOX2 have been included in the manuscript.

2.5 Cell transfection to silence RBFOX2

SW620 and HCT116 cells were seeded respectively at $3x10^4$ or $2.5x10^4$ per cm^2. After 24h, cells were transfected with two different silencerØ Pre-designed siRNA for RBFOX2 (siRNA ID respectively 136602 and 136602 form Life Technologies), or siRNA scramble (scr) as Negative Control. For cell transfection, HiPerFect Transfection Reagent (cat. n±301704, Qiagen, Germany) was used following the manufactureris standard instructions. Eighteen hours after transfection, the cells were processed for the following experiments.

* Additionally, evaluating how LncH19 expression varies under these conditions could enhance understanding of the molecular interactions involved. Moreover, improving the quality of the western blot in FIG 3F would strengthen the visual representation of the experimental results.

Following the revieweris suggestion we investigated lncH19 expression levels. We found that these are not affected by RBFOX2 silencing. This data has been described in the text and added in the new Figure 3.

Moreover, Western blot quality has been improved; we hope this will better represent the experimental results.

* The authors demonstrate an increase in RAC1b expression due to lncH19 overexpression (FIG4A). It would be informative if the authors could provide evidence of lncH19 overexpression in HCT116 and SW620 cell lines. Additionally, improving the quality of the western blot for the HCT116 cell line (FIG4B) would enhance clarity, as currently, there appears to be no noticeable variation in the expression of RAC1b.

Thanks for this suggestion, the increase of lncH19 expression level induced by transfection has been shown in the new Figure 4A and a better-quality western has been shown in the new Figure 4C

* The authors demonstrate an increase in the nuclear localization of RAC1B in lncH19-overexpressing CRC cells (FIG.4C). It would be helpful if the authors clarified the methodology used for immunofluorescence quantitation. Additionally, showing other evidence of nuclear translocation of RAC1B following overexpression or knockdown of lncH19 could strengthen the findings. One approach could be to separate the nuclear and cytoplasmic fractions of the HCT116 and SW620 cell lines and assess the expression of RAC1B in each fraction using either Western blotting or qPCR.

We apologize for the lack, the methodology used for immunofluorescence quantification has been added in material and methods, in particular:

Quantification of the RAC1b signal has been performed using NIS-Elements software. We used a confocal microscope to capture the nuclear plane of each field. Using software, we identified the nuclei as Regions of Interest (ROI) based on the Hoechst signal. The software then calculated the intensity of the signal in the green channel (RAC1b) within each ROI, providing a measure of the mean fluorescence intensity (MFI) relative to the nuclear region. We analysed three different fields per condition. The experiments have been performed in triplicate.

Moreover, we performed a western blot from the nuclear extracts that confirmed the data.

Western blot and relative densitometric analyses have been added in the new Figure 4E, described in the results and in material and methods.

* The authors demonstrated that following overexpression of LncH19, the expression of c-myc and cyclin D increases (FIG.4D). It would be valuable for the authors to evaluate whether this increase in gene expression correlates with changes in cell cycle progression or proliferation in both cell lines.

Following the reviewer's suggestion we investigated the cell cycle in our cellular models. The graph below shows that already 24 hours after lncH19 over-expression, a slight shift of the cell cycle in favour of the G2/M phase is observed, this is impeded by EHT treatment. This data has been introduced in the revised manuscript as Supplementary Figure 5

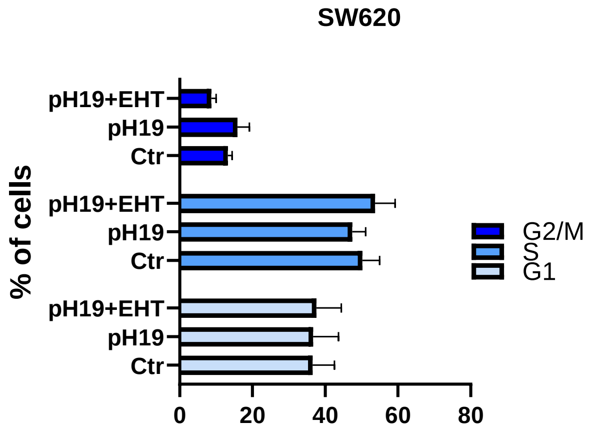

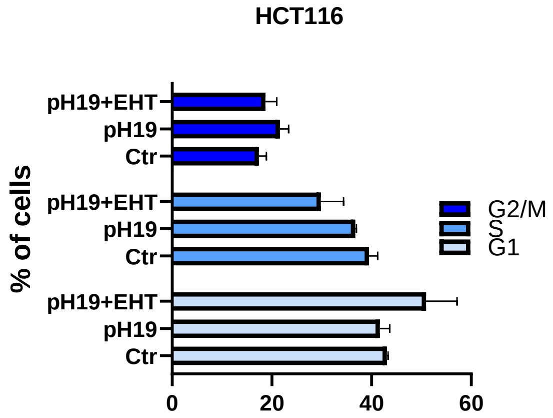

* The in vivo experiments may not be sufficient to demonstrate the direct relationship between lncH19 and RAC1B, as there is no significant increase in RAC1B expression in tissues with higher lncH19 expression. Additionally, previous research has already shown an increase in lncH19 expression in CRC tumor tissue in vivo (doi: 10.1186/s13046-020-01619-6). Therefore, focusing on in vitro experiments could be more informative. For example, the authors could assess the ability of HCT116 and SW620 cell lines to invade, migrate, and induce epithelial-mesenchymal transition (EMT) following lncH19 silencing. They could then determine whether this activity is attenuated or accentuated following silencing of RAC1B. This approach would help elucidate whether RAC1B contributes to the invasion of colorectal cancer cells induced by lncH19.

We thank the reviewer for the interesting suggestion. By gain and loss of expression has been largely demonstrated that lncH19 affects CRC cell EMT and migration, the most recent data are collected in this review (doi: 10.59249/TDBJ7410) however, the molecular mechanism by which lncH19 affects colorectal cancer seems to be more complicated than expected.

Following the reviewer's suggestion, by using RAC1B inhibitor in cells with lncH19 overexpression, we investigated to what extent RAC1B induction could mediate H19-induced phenotypic change.

Firstly, we conducted a transcriptional investigation for the EMT master regulators Snail and E-Cadherin, but we did not observe significant changes in gene expression (see the graph below).

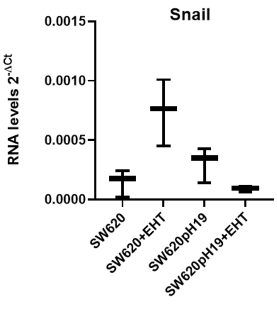

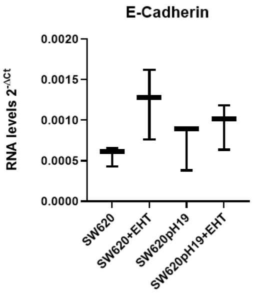

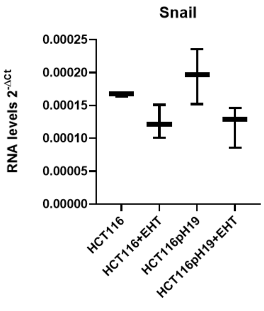

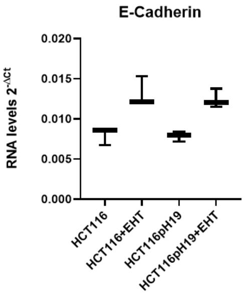

More interesting is the protein analysis for the same marker (see below the graph obtained by densitometric analyses). Here it is possible to appreciate that the RAC1B inhibitor works in opposition to lncH19 expression.

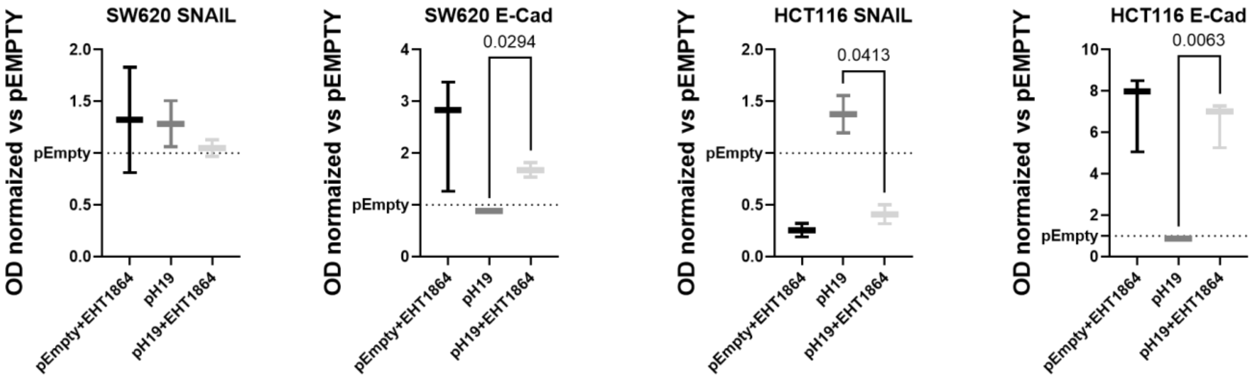

Finally, the figure below shows scratch assay in the same conditions described before.

As expected, the over-expression of lncH19 enhanced cell motility in CRC cell lines, interestingly this effect has been counteracted by the treatment with RAC1B inhibitor.

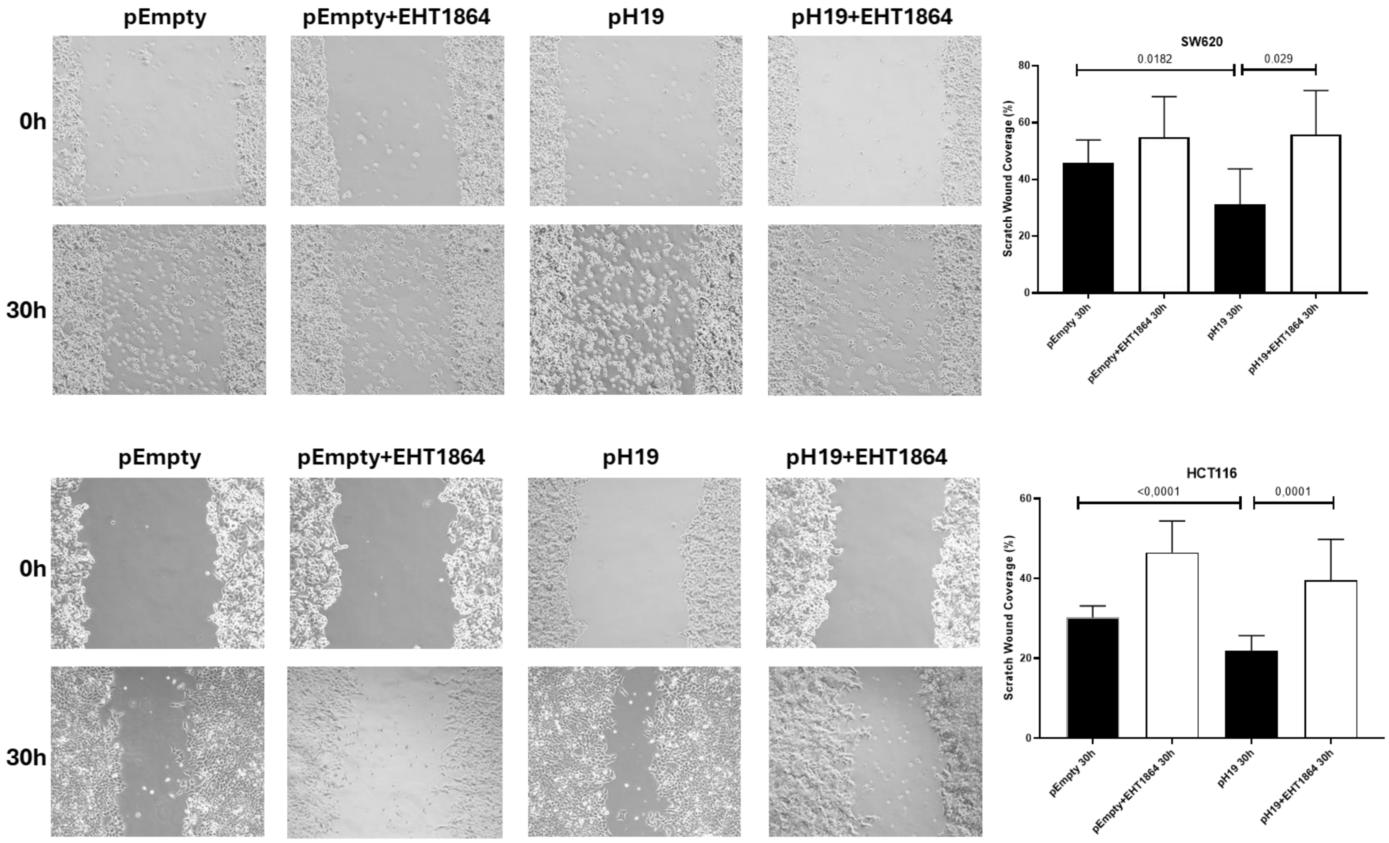

These data are very promising; however, it is important to note that RAC1 inhibition per se shows anti-EMT activityProbably, clearer results could be obtained by using non-tumoral cells or less aggressive cells that do not express RAC1B or lncH19. We aim to better define and characterize this aspect so hope that the reviewer can understand our decision to exclude this

new data from the manuscript to have the possibility to present these in another work supported by more specific molecular data.

* In Figure 6, the authors demonstrate how the binding between RAC1 and RAC1b to splicing factors varies following silencing of LncH19. However, the standard deviations are very high. Improving the statistical analyses could enhance the reliability of the results.

Thanks to this careful observation, a subsequent replication allowed us to enhance the statistical analyses for the Fig 6A right panel and Figure 6D left panel. No variation was observed for the other conditions. Please see the new Figure 6 in the manuscript.

Minor points:

* In Figure 1C, the second graph contains an error on the y-axis. The immunoprecipitation is for hnRNPM, not RBFOX2.

We thank the reviewer for his careful observation. We have corrected the error on the y-axis of the Fig1C

* In Figure 2E, the gene whose expression is evaluated is RAC1B, not RAC1

We apologize for this inaccuracy, and we have corrected RAC1 in RAC1B on the Figure 2

* In Figure 3H, the cell type in which lncH19 has been silenced is HCT116, not SW620.

We apologize for the inaccuracy, and we have corrected Figure 3H which is now the new Fig. 3L.

* In paragraph 3.2 of the results when discussing the silencing of lncH19 for CRC cell lines, it refers to the wrong figures: (FIG.3 G-I). It should be FIG.3 G,H.

We thank the reviewer for his careful observation. We corrected this mistake according to the new Figure 3 thus when we discussed the lncH19 silencing, it refers to Fig. 3I-M

* In figure 6B and D, the graphs contain errors on the y-axis: "hnRPM" instead of "hnRNPM", and in the last graph, the authors should write "hnRNPM" instead of "RBFOX2" on the y-axis.

We apologize for this inaccuracy, and we have corrected the errors on the y-axis of the Figure 6B and D.

* In paragraph 3.3 of the results, when the authors assert that in CRC cell lines HCT116, RAC1B migrates into the nucleus and, recruited at the promoters of Wnt target genes c-Myc and CiclynD1, enhances their expression, a citation is lacking. The authors could cite (doi: 10.3892/ijo.2011.1066), which was mentioned in the discussion [28].

Following the Reviewer's suggestion we added the lacking citation in the main text by mentioning doi: 10.3892/ijo.2011.1066.
